# GEMINI: a variational Bayesian approach to identify genetic interactions from combinatorial CRISPR screens

**DOI:** 10.1186/s13059-019-1745-9

**Published:** 2019-07-12

**Authors:** Mahdi Zamanighomi, Sidharth S. Jain, Takahiro Ito, Debjani Pal, Timothy P. Daley, William R. Sellers

**Affiliations:** 1 Broad Institute of MIT and Harvard, Cambridge, 02142 USA; 2 Department of Statistics, Stanford University, Stanford, 94305 USA; 3 Department of Bioengineering, Stanford University, Stanford, 94305 USA; 4 Deparment of Medical Oncology, Dana-Farber Cancer Institute, Boston, 02115 USA; 5 Department of Medicine, Harvard Medical School, Boston, 02115 USA

**Keywords:** GEMINI, CRISPR, Combinatorial, Double knockout, Genetic interactions, Variational inference, Synthetic lethality

## Abstract

Systems for CRISPR-based combinatorial perturbation of two or more genes are emerging as powerful tools for uncovering genetic interactions. However, systematic identification of these relationships is complicated by sample, reagent, and biological variability. We develop a variational Bayes approach (GEMINI) that jointly analyzes all samples and reagents to identify genetic interactions in pairwise knockout screens. The improved accuracy and scalability of GEMINI enables the systematic analysis of combinatorial CRISPR knockout screens, regardless of design and dimension. GEMINI is available as an open source R package on GitHub at https://github.com/sellerslab/gemini.

**Electronic supplementary material:**

The online version of this article (10.1186/s13059-019-1745-9) contains supplementary material, which is available to authorized users.

## Background

A genetic interaction can be defined as a combination of genetic alterations that leads to an unforeseen loss or gain of cell viability [1]. Genetic interactions have been studied in model systems to elucidate the functional relationships within pathways and important cellular processes [2–4]. In addition, genetic interactions have been leveraged to treat a variety of cancer subtypes in humans [5]. For example, BRCA mutant cancers are specifically vulnerable to PARP inhibition [6]. Similarly, in *BRAF* mutant cancers, combined inhibition of BRAF with other members of the MAP kinase family, such as *MAPK1*-*MAPK3* and *MAP2K1*-*MAP2K2*, overcomes emergent mechanisms of resistance [7].

Although genetic interactions have been systematically characterized in yeast, comprehensive studies in human cells have not been feasible until recently [8]. This is in part due to the large number of gene combinations (roughly 10 times larger than yeast), functional redundancy between and within gene families, and the context specificity of interactions across many distinct cell types. Recent efforts have enabled high-throughput interrogation of genetic interactions in human cells through combinatorial CRISPR-Cas9 screens [9–13]. The analysis and interpretation of these screens, however, remains challenging due to notable variation in guide activity, high replicate variability, and differences in library design.

While existing algorithms have captured some of the strongest interactions in combinatorial screens, they fail to identify many that have been well-characterized. These methods often assume a deterministic relationship between guide or gene pairs and consider replicates, samples, or reagents independently without accounting for the inherent variability and interdependence in combinatorial screens. Moreover, each method has been developed for a specific library format, and as combinatorial screens continue to expand and improve, there remains a strong need for a scalable and systematic method to discover genetic interactions from a variety of screen designs.

Here, we introduce GEMINI, a variational Bayes approach to systematically infer genetic interactions from pooled combinatorial CRISPR knockout screens of any structure. GEMINI infers both reagent- and gene-level effects using data from all samples, replicates, and reagents simultaneously. Our probabilistic approach accurately infers effects attributed to single genes and models a “combination” effect that captures any phenotype that cannot be explained by the addition of two individual gene effects. To account for the various definitions of genetic interactions, we define multiple scoring systems that relate the individual and combination effects to identify interactions exhibiting patterns of synthetic lethality and recovery. We demonstrate a significant improvement over current methods in identifying genetic interactions and show broad applicability to a variety of combinatorial CRISPR knockout screens.

## Results

### Overview of GEMINI

**Fig. 1:**
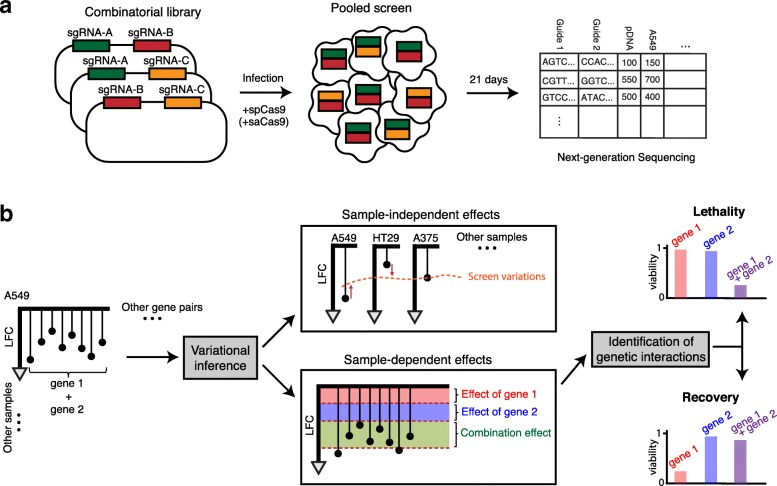
The GEMINI framework for identification of genetic interactions from pairwise CRISPR knockout screens. **a** Combinatorial CRISPR libraries are designed using unique guide pairs to target two genes. The library is then screened in a pooled format, and next-generation sequencing is performed to obtain guide pair representations at early and late time points. **b** Observed log-fold changes (LFCs) of guide pairs are used to infer effects across all samples (sample-independent) and effects within each sample (sample-dependent). Sample-dependent effects are then used to score and identify significant genetic interactions

GEMINI is designed to infer genetic interactions from pooled loss-of-function pairwise CRISPR knockout screens across any number of samples (Methods and Supplementary Methods in Additional file 1). A typical pooled pairwise CRISPR viability screen involves the introduction of Cas9 derived from *Streptococcuspyogenes* into mammalian cells, followed by the introduction of large lentiviral libraries of sgRNA pairs (Fig. 1a). In some designs, an independent Cas9 from *Staphylococcusaureus* is introduced as well [13]. During cell growth, stably transduced sgRNA pairs that lead to a loss of viability are depleted from the cell population while those that enhance viability increase in abundance. The abundance of each sgRNA at the end of the experiment is determined by next-generation sequencing.

GEMINI begins by calculating the log-fold changes (LFCs) in sgRNA pair abundance between an early time point (e.g., plasmid DNA) and a later time point (e.g., day 21 post-infection) and then models the observed LFC as a function of sample-independent and sample-dependent effects (Fig. 1b). Sample-independent effects capture systematic screen variation, including CRISPR guide (i.e., sgRNA) activity, promoter strength, batch effect, or other sources of variation across screens that might otherwise confound efforts to identify genetic interactions. Sample-dependent effects include individual gene effects, which are the phenotypes resulting from single gene perturbation, and a combination effect, namely the phenotype resulting from an interaction between two genes.

To infer sample-independent and sample-dependent effects, we employ a coordinate ascent variational inference (CAVI) approach [14], for which closed-form coordinate updates are obtained analytically to streamline GEMINI’s iterative process. In a typical screen, each guide is paired with at least one biological negative control guide, which GEMINI uses to initialize the effect of an individual gene to the median LFC of guide pairs that target both the gene and a negative control. In the absence of negative controls, GEMINI instead uses the median LFC of guide pairs targeting the gene paired with all other genes to initialize the individual gene effect. The parameters are then updated with information from all samples and all guide pairs simultaneously until convergence. Finally, the inferred effects are used to score the relative strengths of lethality and recovery interactions in each sample, using two scoring systems. The “strong” system captures genetic interactions with high synergy, where the combination effect of an interaction is much greater than either of the individual effects. This can also be thought of as identifying interactions with phenotypes that are “much more than additive.” The “sensitive” measure captures interactions where the phenotype of the pair provides either increased lethality or recovery compared to the individual phenotypes. We define a lethality interaction such that loss of either gene does not lead to a significant decrease in viability whereas loss of both genes does so. We define a recovery interaction such that loss of one gene or the other leads to a decrease in viability and loss of both restores viability. Given known non-interacting gene pairs, GEMINI can also compute Benjamini-Hochberg-corrected false discovery rates [15] (FDRs) to determine significant lethality and recovery interactions, using a stringent threshold of FDR <0.01.

### Performance evaluation

We primarily evaluated our method using data from the Big Papi SynLet library, a combinatorial CRISPR knockout library in which 25 genes and 4 controls were targeted in all possible pairwise combinations to identify synthetic lethal interactions [13]. This dataset includes a set of gene pairs that have been studied in orthogonal screens [16], allowing for an unbiased assessment of GEMINI. Additionally, the library was screened in 6 unique cell lines, enabling the identification of context-specific and context-independent interactions. The Big Papi SynLet library, as well as other combinatorial knockout libraries [10–12], sought to investigate genetic interactions within particular gene families and were tailored to find synthetic lethal interactions. Because of this inherent bias, we present results on lethality scores. However, our method is also capable of identifying interactions that rescue viability (in Additional file 1: Supplementary Information and Figure S1 and in Additional file 2: Table S1).

#### Identification of essential genes and lethal interactions

**Fig. 2:**
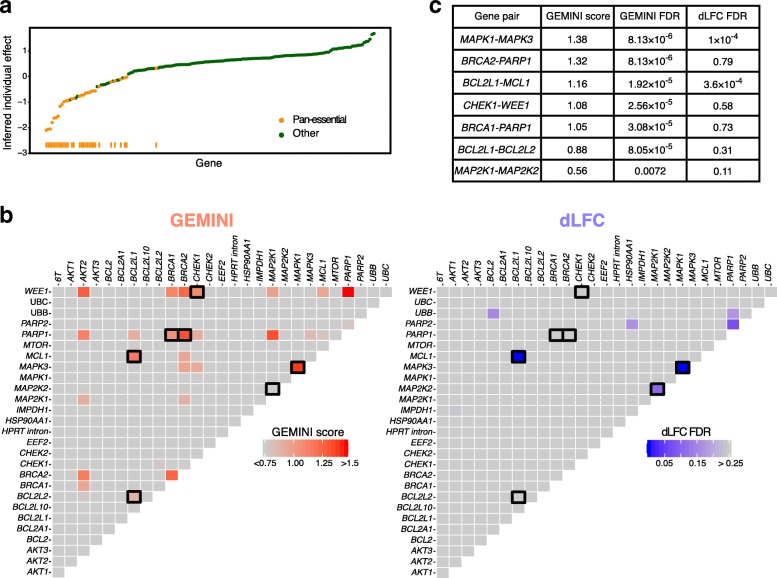
GEMINI accurately infers individual gene effects and identifies strong interactions. **a** GEMINI’s individual gene effects for all of Big Papi’s cell lines are shown. Genes (*x*-axis) are sorted in ascending order according to the inferred individual gene effects (*y*-axis). Essential genes found by CERES [17] are labeled as “Pan-essential.” **b** GEMINI “strong” scores (left) versus dLFC FDRs (right) for all gene pairs in the A549 lung cancer cell line from the Big Papi screen. Pairs with GEMINI score above 1 have FDR less than 0.0001 (see Additional file 2: Table S1 for GEMINI’s scores and FDRs). Experimentally or clinically supported interactions are highlighted in black. **c** GEMINI score, GEMINI FDR, and dLFC FDR for interactions shown in black in part **b**. dLFC does not find the well-known interactions between BRCA family members and *PARP1* while GEMINI finds them highly significant

The individual gene effects inferred by GEMINI for the six cell lines in the Big Papi SynLet screen are shown in Fig. 2a. We observe that the majority of individual effects exhibiting a dropout in LFC are associated with genes that are characterized as essential for viability in whole-genome single gene knockout CRISPR screens using CERES [17]. Moreover, the individual gene effects were highly correlated with CERES scores, with a high average Pearson correlation across all cell lines (*r*=0.83, in Additional file 1: Figure S2).

We next calculated the “strong” interaction score that compares the inferred combination effect to the individual gene effects to determine the strength of a genetic interaction (“Methods” section). GEMINI identified context-specific interactions such as *MAPK1*-*MAPK3*, which was found as a strong lethal interaction only in *RAS*/*RAF* mutated lines. The highest scoring cell line for *MAPK1*-*MAPK3* was A549 (“strong” interaction score =1.38 and FDR =8.13×10^−6^), a lung cancer cell line harboring a KRAS^G12S^ allele (Additional file 2: Table S1). Here, we used gene pairs involving negative controls to compute FDR (Supplementary Methods in Additional file 1). GEMINI identified additional genetic interactions in A549 cells that are supported by findings in the literature (Fig. 2b, c). Normalized read counts for these interactions are shown in Additional file 1: Figure S3. In particular, we observed strong interactions between the anti-apoptotic genes *BCL2L1*, *BCL2L2*, and *MCL1* [18], as well as the synthetic lethal relationship between the BRCA family genes and *PARP1* [6]. Although we detect more well-known genetic interactions compared to the original approach taken in Najm et al. [13] (dLFC), we note that the *MAP2K1*-*MAP2K2* relationship [19] identified by dLFC in the A549 cell line was not strongly significant in GEMINI. This is likely due to the fact that, despite an overwhelming majority of guides suggesting no interaction, dLFC is highly sensitive to a single guide pair displaying a strong phenotype (Additional file 1: Figure S4).

Overall, our findings suggest that GEMINI can accurately infer individual and combination gene effects and determine interactions that are specific to cellular contexts. In order to more comprehensively evaluate GEMINI, we next focused on lethal interactions found across cell lines, with the notion that these interactions are more likely to be context-independent and therefore in agreement with other studies that have characterized synthetic lethal interactions in various contexts.

#### Comparison with previous methods

**Fig. 3:**
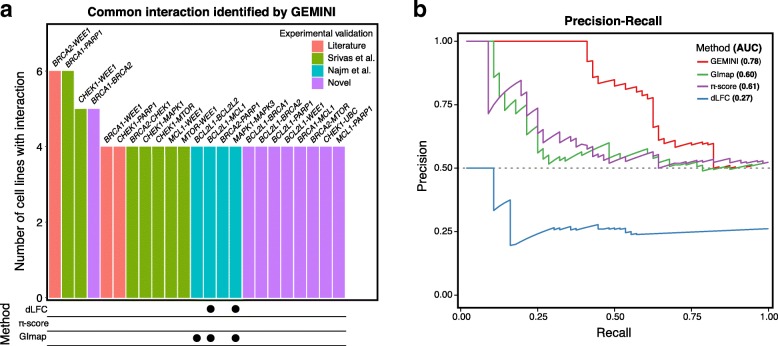
Systematic evaluation of GEMINI. **a** Lethal interactions that were identified by GEMINI in at least four cell lines (Big Papi), colored according to previous findings. Blue indicates interactions validated in Big Papi, green indicates interactions identified by a drug-RNAi screen, red indicates interactions studied in other clinical or experimental contexts, and purple indicates interactions uniquely found by GEMINI that have not been experimentally characterized. Only 3 interactions are found in 4 or more cell lines by other methods (bottom), representing significantly lower sensitivity compared to GEMINI. **b** PR curve is depicted for GEMINI, *π*-score, GImap, and dLFC, where GEMINI achieves the highest PR-AUC (0.78)

To compare GEMINI performance to other methods, we focused on lethal interactions identified in more than half of the six cell lines screened in Big Papi. We computed the “sensitive” interaction score that measures the strength of the total depletion of a gene pair compared to the individual gene effects (“Methods” section). We used this more sensitive approach compared to the “strong” interaction score to capture interactions that show any modest synergy, as compared to the “strong” interaction score that requires a high magnitude of synergy. Gene pairs with significant lethal interaction scores (FDR <0.01) in more than three cell lines are depicted in Fig. 3a. Normalized read counts for these interactions are shown in Additional file 1: Figure S5. GEMINI captured 23 interactions of which 12 agreed with findings from biochemical assays and clinical studies [13, 16, 20, 21]. We also assessed the performance of alternative methods that have been recently utilized to analyze pairwise CRISPR knockout screens, namely dLFC [13], GImap [22], and *π*-score [10]. Here, *π*-score generated *z*-scores and GImap scores approximately followed a normal distribution and thus were transformed to *z*-scores, and FDR was used for dLFC due to its rank-based approach (detailed analysis in Supplementary Information in Additional file 1). Lethal interactions with *z*-scores <−2 or FDR <0.1 in at least four cell lines were considered significant. Noting that the majority of interactions identified by GEMINI were experimentally validated, these methods only found three of the interactions (Fig. 3a, bottom).

To perform a more systematic comparison, we established a set of “true positive” interactions by considering relationships within functional groups [13] to be true and included other identified relationships from a previously established network of conserved lethal interactions [16], resulting in 56 true positive interactions (Supplementary Information in Additional file 1). Interactions involving the negative control genes *HPRT intron* and *6T* were used as a “true negative” set (51 true negative interactions), and *CD81* was provided as a negative control gene to all methods. From these definitions, we computed precision-recall (PR) and receiver operator characteristic (ROC) curves for GEMINI, dLFC, GImap, and *π*-score, where interactions found in at least four cell lines were considered as positive predictions. To enable a fair comparison, we transformed our “sensitive” interaction score to *z*-score since GEMINI requires the “true negative” set to calculate FDR. Thus, *z*-score was used for all methods, except for dLFC for which FDR was used. GEMINI achieved an area under the precision-recall curve (PR-AUC) of 0.78, while *π*-score, GImap, and dLFC achieved 0.61, 0.60, and 0.27, respectively (Fig. 3b). The area under the receiver operator characteristic (ROC-AUC) for GEMINI was at least 0.15 higher compared to these methods (Additional file 1: Figure S6). We also observed that GEMINI consistently outperforms existing methods in identifying interactions common across varying numbers of cell lines (Additional file 1: Figure S7).

#### Impact of the number of guides and cell lines on performance

We examined the performance of GEMINI under varying numbers of guide pairs and samples. We randomly down-sampled from the Big Papi dataset 20 times, selecting 2 guide pairs per gene pair. We increased the number of guide pairs from 2 to 18 incrementally and generated 20 datasets for each specific number of pairs, resulting in a total of 340 datasets. We were not able to run *π*-score and GImap on these datasets since the implementations of these methods were specifically designed for symmetric screens that cover all guides in combination. Moreover, dLFC performed worse than random chance in the complete dataset (Additional file 1: Figure S6) and was thus excluded from further assessment. GEMINI was applicable to all tested datasets and was robust to variations in guide pair and sample numbers. Specifically, using the same procedure performed in the method comparison, we observed that GEMINI achieves higher PR-AUCs and ROC-AUCs as the number of guide pairs increases (Additional file 1: Figure S8). For instance, PR-AUC and ROC-AUC improved by approximately 0.15 each when the number of guide pairs increased from 2 to 18.

We next investigated the extent to which GEMINI benefits from the joint analysis of samples. We emphasize that other methods can only be applied to single samples or replicates. GEMINI was run individually on each sample, and then jointly on all samples. Joint analysis of the data better identified interactions, resulting in an increase of PR-AUC and ROC-AUC by 0.07 and 0.10, respectively (Additional file 1: Figure S9).

#### Impact of library design on performance

Recognizing that the Big Papi library covers a small subset of genes and is designed differently from other existing combinatorial screens, we also evaluated our approach using data from CDKO [11], a combinatorial library consisting of 490,000 guide pairs that targets 21,321 gene pairs. This library includes 207 genes in an all-by-all format, only uses *S. pyogenes* Cas9, and was screened in K562 leukemia cells. Since there is no comprehensive validation for interactions in CDKO to construct an unbiased true positive set, we used SynLethDB [23], a extensive database that contains synthetic lethal pairs identified experimentally and computationally across model organisms. Treating SynLethDB interactions as context-independent, we found 90 synthetic lethal pairs common between SynLethDB and CDKO, which we used as a true positive set. We also randomly divided the 79 non-targeting negative control guides in CDKO into group 1 with 39 guides and group 2 with 40 guides, where group 1 was used as a negative control and group 2 was treated as a gene that should not synergize with other genes, resulting in a set of 207 true negative pairs. Given these validation sets, we calculated the ROC-AUC and PR-AUC for GEMINI, GImap, and *π*-score (*z*-scores calculated for all methods). This was performed analogously to our previous assessment using Big Papi, but treating any interactions found in K562 as positive predictions. We could not run dLFC as there is no source code available and only its final results are reported for Big Papi. GEMINI achieved an ROC-AUC of 0.79 and PR-AUC of 0.80, while GImap’s values were 0.71 and 0.68, and *π*-score’s values were 0.49 and 0.32 (Additional file 1: Figure S10). We have shown that GEMINI performance improves as the number of cell lines increases (Additional file 1: Figure S9). Thus, we expect that GEMINI performance will show continued improvement over existing methods in large libraries screened across multiple cell lines.

Although all published combinatorial screens include biological negative controls that are paired with all other guides in the library, we also assessed GEMINI performance in the case where no negative control is specified. Alternative methods are all dependent on the inclusion of negative control guides and thus are not applicable for this assessment. In the absence of negative control to initialize the individual gene effects in CAVI approach, GEMINI uses the median LFC of all guide pairs targeting one gene paired with all other genes. We emphasize that such initialization is not recommended for non-all-by-all screen designs due to the limited number of gene pairs including each gene. In the case of Big Papi, we compared the results based on this initialization to those achieved when a negative control was given. Using the validation set described previously for Big Papi, we observed that GEMINI consistently performs better when a negative control is available. Specifically, the ROC-AUC and PR-AUC values are 0.15 and 0.1 lower if a negative control is not provided (Additional file 1: Figure S11a). Using the validation set described previously for CDKO, we observed a less of a decrease in performance (0.05 and 0.07 in ROC-AUC and PR-AUC, in Additional file 1: Figure S11b), but still saw consistent outperformance using negative control. This is mainly due to the large size of CDKO screen, allowing GEMINI to utilize more observations to improve its estimation of sample-dependent effects.

### Cross-screen analysis

**Fig. 4:**
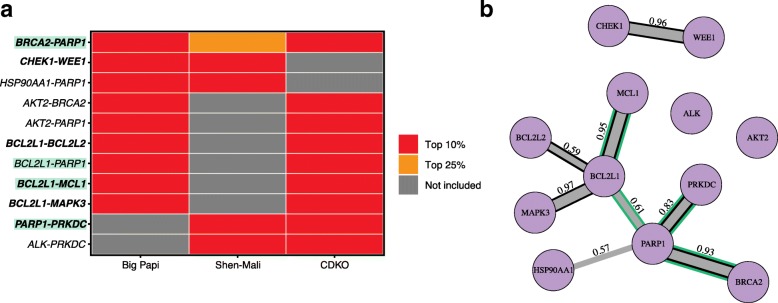
GEMINI identifies interactions common across different combinatorial knockout screens. **a** Common interactions identified by GEMINI across Big Papi, Shen-Mali, and CDKO screens are shown. Any gene pairs in the top 10% of ranks in at least two screens are shown in red. If a gene pair was not included in the screen, it was labeled as “Not included” (gray). Experimentally or clinically supported interactions are shown in bold, and those identified in SynLethDB are colored by green. **b** Common interactions across screens are shown in a network where edge weight is adjusted based on evidence scores from STRING. Only interactions with evidence scores above 0.5 are shown. Interactions supported by experimental or clinical findings are emphasized with black edges, and those supported by SynLethDB with green edges

In addition to Big Papi and CDKO, we applied GEMINI to other publicly available pairwise CRISPR knockout screens, Zhao-Mali [12] and Shen-Mali [10]. Despite differences in data quality and library design across screens, the CAVI algorithm that was implemented in GEMINI rapidly achieved convergence for all screens, with the most drastic changes in mean absolute error occurring within the first 5 iterations (Additional file 1: Figure S12). To further assess the obtained results, we focused on interactions that were found in multiple screens. We calculated the “strong” interaction score for all gene pairs in each screen and selected the strongest interaction scores across all cell lines if the screen was performed in more than one cell line. We then ranked gene pairs according to these scores and identified the top 10% ranked gene pairs found in two or more screens (Fig. 4a). Note that only 190 gene pairs were in common across any two screens while Zhao-Mali had no pairs in common with other screens and thus was not considered in this analysis. Across all three screens, the well-known *BRCA2*-*PARP1* interaction was identified [6]. Overall, GEMINI found 11 interactions of which 7 were previously experimentally validated [6, 13, 21, 24, 25] or previously identified as a synthetic lethality in SynLethDB [23]. We also evaluated these interactions using STRING [26], a database of known and predicted protein-protein interactions. We retrieved STRING evidence scores of the 11 interactions (between 0 and 1), which reflects the likelihood of an interaction being biologically true given experimental and computational evidence. Recognizing that STRING might have high levels of false positives, we found 8 interactions (out of 11), with the evidence score above 0.5 (Fig. 4b). This list includes all the 7 interactions that were highlighted in Fig. 4a. Our findings indicate that GEMINI is applicable to various screen formats and enables a systematic integration of combinatorial CRISPR knockout screens for the discovery of novel genetic interactions.

## Discussion

### Reagent variability

Although GEMINI accounts for different sources of variation in combinatorial CRISPR knockout screens and improves upon existing methods, we observed certain patterns of variation in guide activity that might lead to incorrect inferences. In particular, if guides targeting a gene pair showed extreme inconsistency (i.e., high reagent variability), GEMINI’s estimate of individual and combination gene effects became less reliable (Supplementary Information, Figure S13, and Figure S14 in Additional file 1). In the two screens with the highest biological replicate correlation, CDKO and Big Papi, we observed such reagent variability in 14.87*%* and 13.33*%* of the most significant hits in each screen (top 5*%*), respectively. For these high-variability pairs, the standard deviation of LFCs for guides targeting each gene pair was, on average across cell lines, greater than 1.

Previous studies suggest that the observed reagent variability might correspond to the properties of guide sequence and the functional impact of a guide on the genetic product rather than a systematic variation that can be captured across samples [27, 28]. If the variation is characterized before the analysis of a combinatorial screen, this information can be used as a prior in GEMINI to downweight or upweight guides and improve performance (Supplementary Information, Figure S15, and Figure S16 in Additional file 1). In the absence of a detailed understanding of reagent variability, we recommend the use of the default parameters that assume a modest confidence on all guide activity.

### Copy number variation

Previous studies have corrected for copy number variation to improve the estimation of gene effects in single knockout CRISPR screens [17, 29]. However, in the case of dual knockout CRISPR screens, published datasets are limited both by the number of genes and cell lines with copy number alterations, and therefore, it is not possible to systematically evaluate any copy number effect in combinatorial screen. In anticipation of larger datasets, pre-processing methods designed for single knockout screens [30, 31] can be generalized to combinatorial screens to remove this effect from LFCs. GEMINI will be able to use these corrected LFCs as an input, without any need for modifications to the inference and scoring procedures.

### Application to other pairwise screens

In evaluating GEMINI’s broader utility, we applied our method to other dropout-based pairwise screens. In particular, we assessed GEMINI’s performance in a pooled combinatorial CRISPRi dropout screen [22]. Although GEMINI identified the majority of interactions described by the authors, we suspect that the predicted interactions include many false positives (Additional file 2: Table S1). This is likely due to an exacerbation of the previously described reagent variability that we observe at a significantly higher rate in combinatorial CRISPRi screens. Specifically, we found that among the top 5*%* of synergistic hits in the CRISPRi screen, 34.61*%* of gene combinations showed significant variability (standard deviation >1). We envision that a better understanding of reagent variability could, in addition to improving GEMINI performance, increase the breadth of applicability of our probabilistic approach beyond knockout screens.

## Conclusions

The emergence of combinatorial CRISPR screening technology has provided new tools to uncover novel genetic interactions and therapeutic opportunities in disease. We developed GEMINI to jointly analyze the output from pairwise knockout screens to identify genetic interactions, while accounting for variability in screen format, such as guide activity, promoter strength, and replicate variability. Our probabilistic approach uncovers many well-characterized interactions from these screens that were not otherwise found by existing methods. We also showed that GEMINI is widely applicable to a variety of library formats and that its performance improves as the number of guides or samples increases. Finally, we demonstrated that, given a better understanding of reagent behavior and copy number variation, GEMINI can incorporate this information to improve the identification of genetic interactions.

As the use of combinatorial screening becomes more widespread, we anticipate that, because our approach makes no assumptions about the screen format, researchers can analyze new screens in a variety of formats. For instance, it could be possible to design more efficient libraries to investigate specific genetic interactions without the need for a large all-by-all guide pair library. Moreover, in libraries screened across tens to hundreds of cell lines, GEMINI will integrate information across all samples to better identify genetic interactions and enable the creation of an interaction map to drive target discovery in cancer and other diseases. GEMINI is available as an open-source R package at https://github.com/sellerslab/gemini.

## Methods

### Model

To estimate sample-independent and sample-dependent effects from LFCs (see Supplementary Methods in Additional file 1 for the calculation of LFCs), we formulate the effects in a Bayesian framework and apply a variational inference method. We later explain the scoring and identification of lethality and recovery interactions.

We assume that the observed log-fold changes can be broken down into three latent effects: the individual effects of each guide in a pair and the effect of two guides in combination. All three effects can be further broken down into the product of two effects: sample-independent and sample-dependent.

Specifically, we define the following notation:

$$\begin{array}{*{20}l} & g \text{ and}\, h= \text{genes } {g} \text{ and } {h} \text{ being targeted simultaneously;} \\ & g_{i} \text{ and} \,h_{j} = \text{guide } {i} \text{ and guide } {j} \text{ targeting genes } {g} \text{ and } {h}, \\ &\text{respectively;} \\ & l = \text{one sample; and}\\ & D_{g_{i}, h_{j}, l} = \text{the observed LFC of the guide pair (\(g_{i},h_{j}\))} \\ &\text{in sample } {l}.\vspace*{-6pt} \end{array} $$

We then model the LFC as

$$D_{g_{i}, h_{j}, l} \sim \mathcal{N}(x_{g_{i}}y_{g,l} + x_{h_{j}}y_{h,l} + x_{g_{i},h_{j}}s_{g,h,l}, \tau^{-1}_{g_{i},h_{j},l}). $$

We explain each latent effect, the assumptions, and the priors chosen in detail below.

#### Sample-independent effects

We let *x*_·_ denote the sample-independent effects of guides. For guide pair (*g*_*i*_,*h*_*j*_), there are three sample-independent effects: the effect of guide *g*_*i*_ ($x_{g_{i}}$), the effect of guide *h*_*j*_ ($x_{h_{j}}$), and the effect of the combination ($x_{g_{i},h_{j}}$). The sample-independent effects include any systematic shifts that may exist between two guides, guide efficacy, guide concordance across samples, and other screen variations. We emphasize that *x*_·_ represents a mixture of sample-independent effects and should not be interpreted as guide efficacy. We assume common normal priors on all sample-independent effects, but users can choose to set different priors on each effect.

$$x_{\cdot} \sim \mathcal{N} \big(\mu_{x}, \sigma_{x}^{2} \big). $$

We recognize that $x_{g_{i}, h_{j}}$ can be dependent on $x_{g_{i}}$ and $x_{h_{i}}$, but ignore such dependency since it is not straightforward to model this relationship. We instead let the observed LFCs across many samples predict these variables. By default, we impose a moderate prior, with *μ*_*x*_=1 and *σ*_*x*_=1. However, in the event of low sample sizes (<3 samples), a stronger prior should be specified (e.g., *σ*_*x*_=0.1) to shrink the estimated effects more towards 1 and reduce false positives.

#### Sample-dependent effects

We let *y*_·_ and *s*_·_ denote the sample-dependent individual and combination effects, respectively. These include any context-specific effects that may exist in each sample. We assume normal priors for sample-dependent effects, with a common prior for gene-level effects and a separate prior for the combination effects, but users can choose to set different priors on each effect.

$$y_{\cdot} \sim \mathcal{N} \big(\mu_{y}, \sigma^{2}_{y} \big), \quad s_{\cdot} \sim \mathcal{N} \big(\mu_{s}, \sigma^{2}_{s} \big). $$

In this case, we assume that prior means, *μ*_*y*_ and *μ*_*s*_, are 0 to reflect the expectation that the majority of effects are close to 0. We set $\sigma ^{2}_{y}$ and $\sigma _{s}^{2}$ equal to 10 to reflect limited prior knowledge of sample-dependent effects.

#### Precision of observed data

We introduce $\tau _{g_{i}, h_{j}, l} \sim \Gamma (\alpha _{g_{i}, h_{j}, l}, \beta _{g_{i}, h_{j}, l})$ to model the precision of observed LFC for each guide pair in sample *l*. We assume the prior follows a Gamma distribution and use replicates to estimate the parameters of this distribution. Direct empirical estimation of the prior parameters can be poor in experiments with a small number of replicates. To overcome this challenge in single guide libraries, previous studies [32, 33] suggested a smoothed version of empirical estimates based on all guides in each sample. Such methods could be directly applied to combinatorial screens by treating guide pairs as single guides. On the other hand, recent combinatorial knockout screens [13] generate highly correlated replicates (Pearson correlation ≥0.9, calculated using replicate counts) that lead to small empirical estimates. As a default, we use direct empirical estimation of the prior parameters, but a smoothed estimate of the parameters is also available in GEMINI (see Supplementary Methods in Additional file 1 for details).

We apply coordinate ascent variational inference [14] to infer the posterior distributions of *x*, *y*, *s*, and *τ*. The variational distribution is assumed to factorize over each latent variable, and coordinate updates are obtained accordingly. Details for computing updates are described in Supplementary Methods in Additional file 1.

### Lethality and recovery interactions

To assess interactions, we relate the individual effects to the non-additive effect through two scoring systems: “strong” and “sensitive” lethality and recovery. These proposed definitions of interactions are well-described and have been used to identify genetic interactions in yeast and other model organisms [1]. However, recognizing that the definitions of genetic interactions can widely vary, users can utilize the inferred sample-dependent effects to define new scoring systems.

#### Strong lethality and recovery

To characterize this relationship, we compare the expected values of *s*_·_ to *y*_·_ after gene and gene pair-level inference. The expected values are shown by *s*_·_ and *y*_·_, acknowledging a misuse of notation. For every gene pair in sample *l*, we compute the score

$$|s_{g, h, l}| - \max(|y_{g, l}|, |y_{h, l}|), $$

which reflects the strength of the combination effect (i.e., |*s*_*g,h*,*l*_|) compared to the individual gene effects (i.e., max(|*y*_*g,l*_|,|*y*_*h,l*_|)). In other words, we capture interactions with phenotypes that are “much more than additive” and primarily driven by the combination effect. Larger positive values indicate a stronger lethality if *s*_*g,h*,*l*_ is negative or a stronger recovery if *s*_*g,h*,*l*_ is positive. We note that our strong recovery definition may also capture other classes of positive growth interactions where the individual gene effects do not result in loss of viability. Our definitions of strong lethality and recovery also may not identify cases with a modest difference between gene pair and individual gene effects. We thus introduce the following definitions to capture a wider range of possible lethality and recovery relationships.

#### Sensitive lethality and recovery

Lethality relationships are scored according to

$$\begin{array}{*{20}l} & \min(y_{g, l}, y_{h, l}) - (y_{g, l} + y_{h, l} + s_{g, h, l}) \\ & \text{subject to}\quad y_{g, l} \ \text{and} \ y_{h, l} > c \lambda \end{array} $$

where *c* is the 0.01-quantile of *y* values in sample *l*, and *λ*=0.5 by default. This constraint removes gene pairs of which at least one gene is more than 50% depleted, compared to genes with the strongest depletion in the screen. We can also define *c* in relation to positive control genes, for instance the median depletion caused by known essential genes. The score value compares the total effect of the gene pair (i.e., *y*_*g,l*_+*y*_*h,l*_+*s*_*g,h*,*l*_) to the most lethal individual gene (i.e., *min*(*y*_*g,l*_,*y*_*h,l*_)), with positive values presenting stronger effects. In other words, this will capture the additional dropout that results from a combination of two genes, where the killing effect of the pair cannot be attributed to the knockout of either gene individually. In addition, recovery effects are scored similar to lethality, but in the opposite direction with a constraint that requires at least one gene to independently exhibit notable viability effect.

$$\begin{array}{*{20}l} & y_{g, l} + y_{h, l} + s_{g, h, l} - \min(y_{g, l}, y_{h, l}) \\ & \text{subject to}\quad y_{g, l} \ \text{or} \ y_{h, l} < c \lambda \end{array} $$

If a set of non-interacting gene pairs is known, GEMINI uses this set to define a null distribution for the above scores, and calculates *p* values and FDR for lethality and recovery interactions. Details are explained in Supplementary Methods in Additional file 1.

## Additional files

Additional file 1 **Figures S1-S16.** Supplementary Methods, and Supplementary Information. (PDF 4131 kb)

Additional file 2 **Table S1.** This table contains the results from running GEMINI on four publicly available combinatorial CRISPR screens. (XLSX 13,104 kb)

Additional file 3 Review history. (DOCX 17 kb)

## References

[1] Mani R, Onge RPS, Hartman JL, Giaever G, Roth FP (2008). Defining genetic interaction. Proc Natl Acad Sci.

[2] Wolf JB, Brodie ED, Wade MJ (2000). Epistasis and the evolutionary process.

[3] Boone C, Bussey H, Andrews BJ (2007). Exploring genetic interactions and networks with yeast. Nat Rev Genet.

[4] Collins SR, Miller KM, Maas NL, Roguev A, Fillingham J, Chu CS, Schuldiner M, Gebbia M, Recht J, Shales M (2007). Functional dissection of protein complexes involved in yeast chromosome biology using a genetic interaction map. Nature.

[5] Kaelin Jr WG (2005). The concept of synthetic lethality in the context of anticancer therapy. Nat Rev Cancer.

[6] Helleday T (2011). The underlying mechanism for the PARP and BRCA synthetic lethality: clearing up the misunderstandings. Mol Oncol.

[7] Wagle N, Van Allen EM, Treacy DJ, Frederick DT, Cooper ZA, Taylor-Weiner A, Rosenberg M, Goetz EM, Sullivan RJ, Farlow DN (2014). MAP kinase pathway alterations in BRAF-mutant melanoma patients with acquired resistance to combined RAF/MEK inhibition. Cancer Discov.

[8] Roguev A, Talbot D, Negri GL, Shales M, Cagney G, Bandyopadhyay S, Panning B, Krogan NJ (2013). Quantitative genetic-interaction mapping in mammalian cells. Nat Methods.

[9] Wong AS, Choi GC, Cui CH, Pregernig G, Milani P, Adam M, Perli SD, Kazer SW, Gaillard A, Hermann M (2016). Multiplexed barcoded CRISPR-Cas9 screening enabled by CombiGEM. Proc Natl Acad Sci.

[10] Shen JP, Zhao D, Sasik R, Luebeck J, Birmingham A, Bojorquez-Gomez A, Licon K, Klepper K, Pekin D, Beckett AN (2017). Combinatorial CRISPR–Cas9 screens for de novo mapping of genetic interactions. Nat Methods.

[11] Han K, Jeng EE, Hess GT, Morgens DW, Li A, Bassik MC (2017). Synergistic drug combinations for cancer identified in a CRISPR screen for pairwise genetic interactions. Nat Biotechnol.

[12] Zhao D, Badur MG, Luebeck J, Magaña JH, Birmingham A, Sasik R, Ahn CS, Ideker T, Metallo CM, Mali P (2018). Combinatorial CRISPR-Cas9 metabolic screens reveal critical redox control points dependent on the KEAP1-NRF2 regulatory axis. Mol Cell.

[13] Najm FJ, Strand C, Donovan KF, Hegde M, Sanson KR, Vaimberg EW, Sullender ME, Hartenian E, Kalani Z, Fusi N (2018). Orthologous CRISPR–Cas9 enzymes for combinatorial genetic screens. Nat Biotechnol.

[14] Blei DM, Kucukelbir A, McAuliffe JD (2017). Variational inference: a review for statisticians. J Am Stat Assoc.

[15] Benjamini Y, Hochberg Y (1995). Controlling the false discovery rate: a practical and powerful approach to multiple testing. J R Stat Soc Ser B Methodol.

[16] Srivas R, Shen JP, Yang CC, Sun SM, Li J, Gross AM, Jensen J, Licon K, Bojorquez-Gomez A, Klepper K (2016). A network of conserved synthetic lethal interactions for exploration of precision cancer therapy. Mol Cell.

[17] Meyers RM, Bryan JG, McFarland JM, Weir BA, Sizemore AE, Xu H, Dharia NV, Montgomery PG, Cowley GS, Pantel S (2017). Computational correction of copy number effect improves specificity of CRISPR–Cas9 essentiality screens in cancer cells. Nat Genet.

[18] Eichhorn JM, Alford SE, Sakurikar N, Chambers TC (2014). Molecular analysis of functional redundancy among anti-apoptotic Bcl-2 proteins and its role in cancer cell survival. Exp Cell Res.

[19] Aoidi R, Maltais A, Charron J (2016). Functional redundancy of the kinases MEK1 and MEK2: rescue of the Mek1 mutant phenotype by Mek2 knock-in reveals a protein threshold effect. Sci Signal.

[20] Garcia TB, Snedeker JC, Baturin D, Gardner L, Fosmire SP, Zhou C, Jordan CT, Venkataraman S, Vibhakar R, Porter CC (2017). A small-molecule inhibitor of WEE1, AZD1775, synergizes with olaparib by impairing homologous recombination and enhancing DNA damage and apoptosis in acute leukemia. Mol Cancer Ther.

[21] Yin Y, Shen Q, Zhang P, Tao R, Chang W, Li R, Xie G, Liu W, Zhang L, Kapoor P (2017). Chk1 inhibition potentiates the therapeutic efficacy of PARP inhibitor BMN673 in gastric cancer. Am J Cancer Res.

[22] Horlbeck MA, Xu A, Wang M, Bennett NK, Park CY, Bogdanoff D, Adamson B, Chow ED, Kampmann M, Peterson TR (2018). Mapping the genetic landscape of human cells. Cell.

[23] Guo J, Liu H, Zheng J (2015). SynLethDB: synthetic lethality database toward discovery of selective and sensitive anticancer drug targets. Nucleic Acids Res.

[24] Zarkovic G, Belousova EA, Talhaoui I, Saint-Pierre C, Kutuzov MM, Matkarimov BT, Biard D, Gasparutto D, Lavrik OI, Ishchenko AA (2018). Characterization of DNA ADP-ribosyltransferase activities of PARP2 and PARP3: new insights into DNA ADP-ribosylation. Nucleic Acids Res.

[25] Boucher M. -J., Morisset J, Vachon PH, Reed JC, Lainé J, Rivard N (2000). MEK/ERK signaling pathway regulates the expression of Bcl-2, Bcl-XL, and Mcl-1 and promotes survival of human pancreatic cancer cells. J Cell Biochem.

[26] Szklarczyk D, Franceschini A, Wyder S, Forslund K, Heller D, Huerta-Cepas J, Simonovic M, Roth A, Santos A, Tsafou KP (2014). STRING v10: protein–protein interaction networks, integrated over the tree of life. Nucleic Acids Res.

[27] Doench JG, Fusi N, Sullender M, Hegde M, Vaimberg EW, Donovan KF, Smith I, Tothova Z, Wilen C, Orchard R (2016). Optimized sgRNA design to maximize activity and minimize off-target effects of CRISPR-Cas9. Nat Biotechnol.

[28] Munoz DM, Cassiani PJ, Li L, Billy E, Korn JM, Jones MD, Golji J, Ruddy DA, Yu K, McAllister G (2016). CRISPR screens provide a comprehensive assessment of cancer vulnerabilities but generate false-positive hits for highly amplified genomic regions. Cancer Discov.

[29] Gonçalves E, Behan FM, Louzada S, Arnol D, Stronach EA, Yang F, Yusa K, Stegle O, Iorio F, Garnett MJ (2019). Structural rearrangements generate cell-specific, gene-independent CRISPR-Cas9 loss of fitness effects. Genome Biol.

[30] Iorio F, Behan FM, Goncalves E, Bhosle SG, Chen E, Shepherd R, Beaver C, Ansari R, Pooley R, Wilkinson P (2018). Unsupervised correction of gene-independent cell responses to CRISPR-Cas9 targeting. BMC Genomics.

[31] De Weck A, Golji J, Jones MD, Korn JM, Billy E, McDonald III ER, Schmelzle T, Bitter H, Kauffmann A (2018). Correction of copy number induced false positives in CRISPR screens. PLoS Comput Biol.

[32] Allen F, Behan F, Khodak A, Iorio F, Yusa K, Garnett M, Parts L (2019). JACKS: joint analysis of CRISPR/Cas9 knockout screens. Genome Res.

[33] Li W, Xu H, Xiao T, Cong L, Love MI, Zhang F, Irizarry RA, Liu JS, Brown M, Liu XS (2014). MAGeCK enables robust identification of essential genes from genome-scale CRISPR/Cas9 knockout screens. Genome Biol.

[34] Zamanighomi M, Jain S. R package: gemini (0.99.0). GitHub. 2019. 10.5281/zenodo.3246925.

