# Supplementary material for: GEMINI: a variational Bayesian approach to identify genetic interactions from combinatorial CRISPR screens
Source: Genome Biol. 2019 Jul 12;20:137. doi: 10.1186/s13059-019-1745-9 (PMC6624979; doi:10.1186/s13059-019-1745-9)
Supplement: Supplementary file 1 — Figures S1-S16. Supplementary Methods, and Supplementary Information. (PDF 4131 kb) [file 13059_2019_1745_MOESM1_ESM.pdf]

Supplementary Materials

Contents

1 Supplementary Figures **2**

2 Supplementary Methods **18**
- 2.1 Calculation of LFCs 18
- 2.2 Precision of LFCs 18
- 2.3 Coordinate ascent variational inference 18
- 2.4 FDR calculation 20

3 Supplementary Information **21**
- 3.1 Identification of recovery interactions 21
- 3.2 Constructing true positive and true negative interaction sets 21
- 3.3 Method comparison 22
- 3.4 Reagent variability 22

1 Supplementary Figures

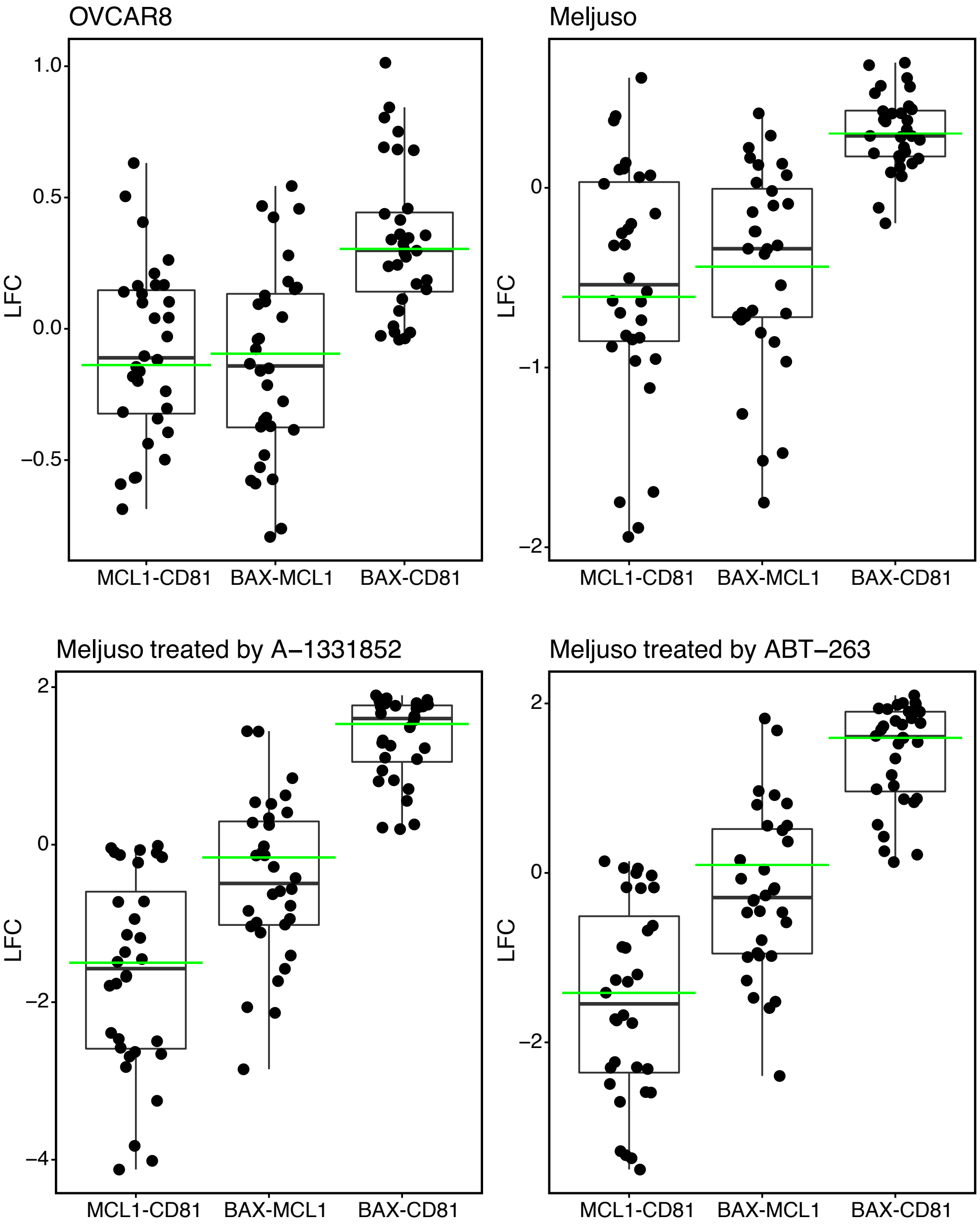

Figure S1: **Identification of recovery interactions in the Big Papi Apoptotic library.** Boxplots indicating the LFCs of guide pairs targeting *MCL1-CD81*, *BAX-CD81*, and *BAX-MCL1*. Each box shows the boundaries of the 25th and 75th percentile values, and the center line indicates the median. The green horizontal line indicates GEMINI's inferred individual and combination gene effects. We use this convention for all boxplots shown in Supplementary Figures. Here, *CD81* is a cell surface marker with no known impact on cell viability, and therefore used as a negative control. The top row shows the effects of *BAX* and *MCL1* knockout in the ovarian cell line OVCAR8 and the skin melanoma cell line Meljuso. The bottom row shows the effects of *BAX* and *MCL1* knockout in Meljuso cells treated with BH3-mimetics. In treated cells, we observe a higher sensitivity to *MCL1* knockout, and significant recovery with the knockout of both genes.

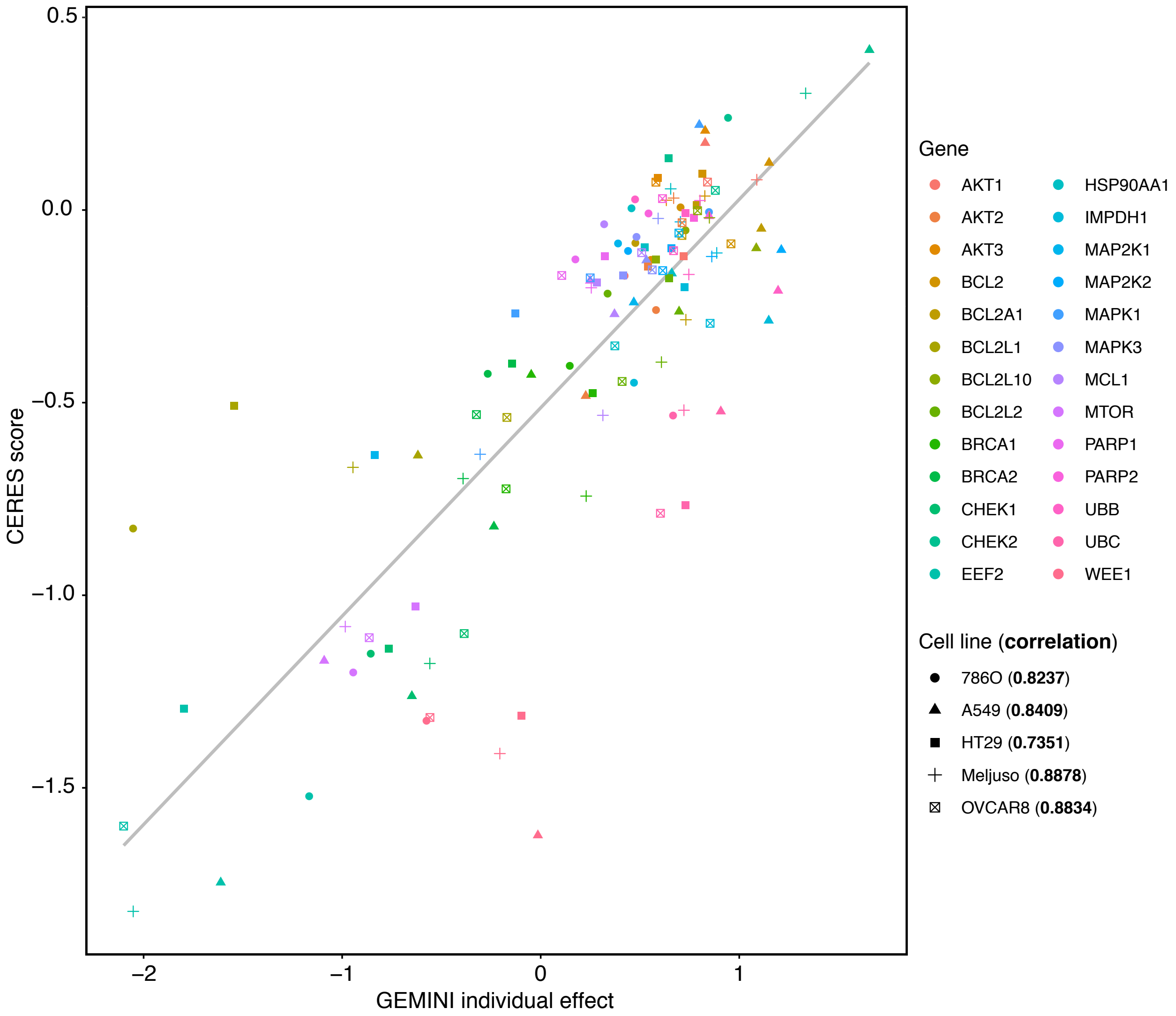

Figure S2: **Individual gene effects are well correlated between GEMINI and CERES.** GEMINI's individual gene effects (i.e. y, see Methods), inferred for the Big Papi dataset, are ploted against CERES gene essentiality scores computed from the Avana library [1]. We note that the data used in Big Papi was derived from the Brunello library, an improved version of Avana. Genes and cell lines are shown by different colors and symbols, respectively. On average, across all cell lines, the Pearson correlation of gene scores between GEMINI and CERES is 0.8342, indicating that GEMINI's results are consistent with the analysis of single-knockout screens. Data for A375 (one of six cell lines in Big Papi) are not shown since this cell line was not screened in the Avana library. In addition, inferred individual effects from CDKO, Shen-Mali, and Zhao-Mali datasets show Pearson correlations of approximately 0.5 to CERES gene effect scores, lower compared to Big Papi, likely due to significant differences in guide selection and library design when compared to Avana.

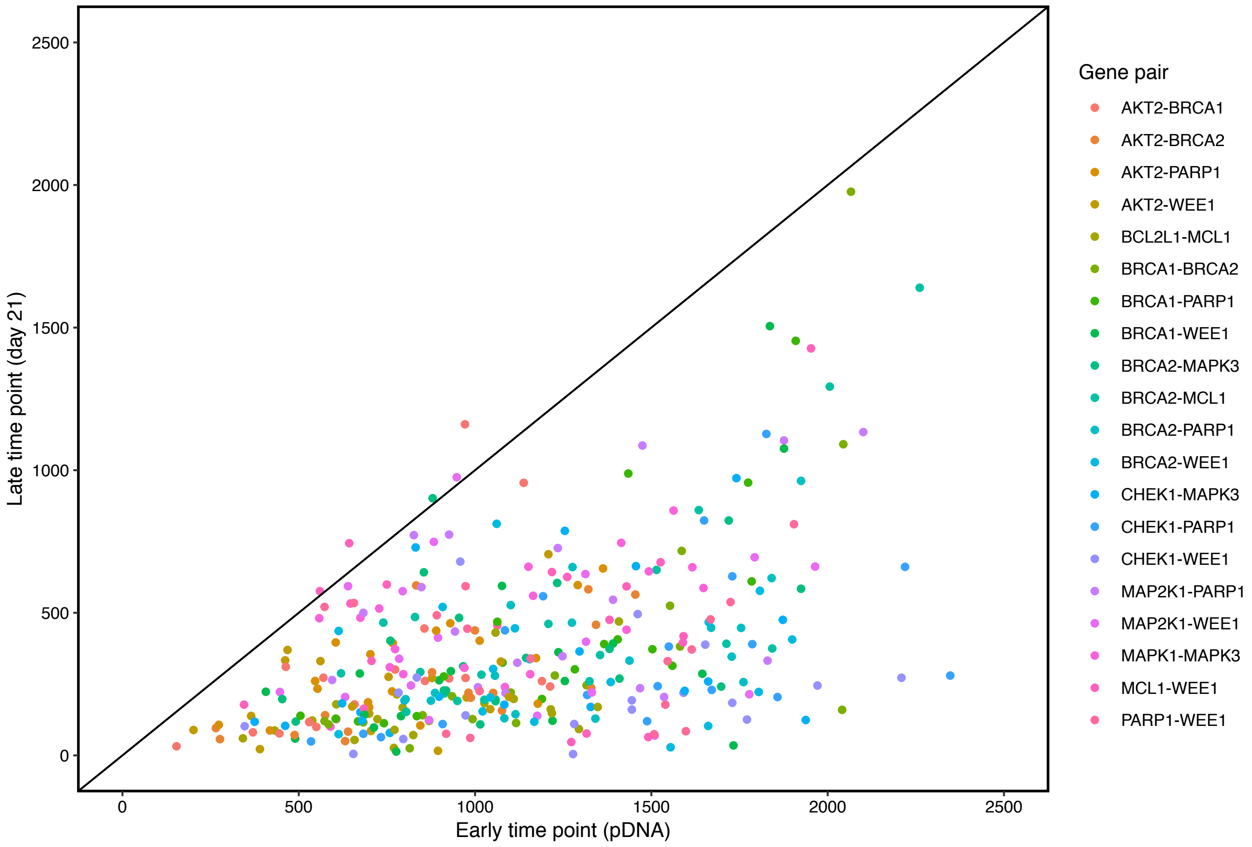

Figure S3: **Normalized read counts for the top 20 interactions in A549 in Big Papi.** We first normalized guide pair counts with respect to the total count across all guide pairs for each cell line, which was then multiplied by a constant (10^7) and averaged across all replicates. Here, we show the normalized counts of early versus late time point for guide pairs corresponding to the top 20 interactions identified by GEMINI in A549 from Big Papi. Guide pairs targeting the same gene pair are shown with the same color. We observe dropout in counts at late time point for almost all guide pairs, suggesting that interactions identified by GEMINI in the Big Papi dataset are in agreement with the raw data. Note that all guide pairs have at least 92 or more raw counts at early time point.

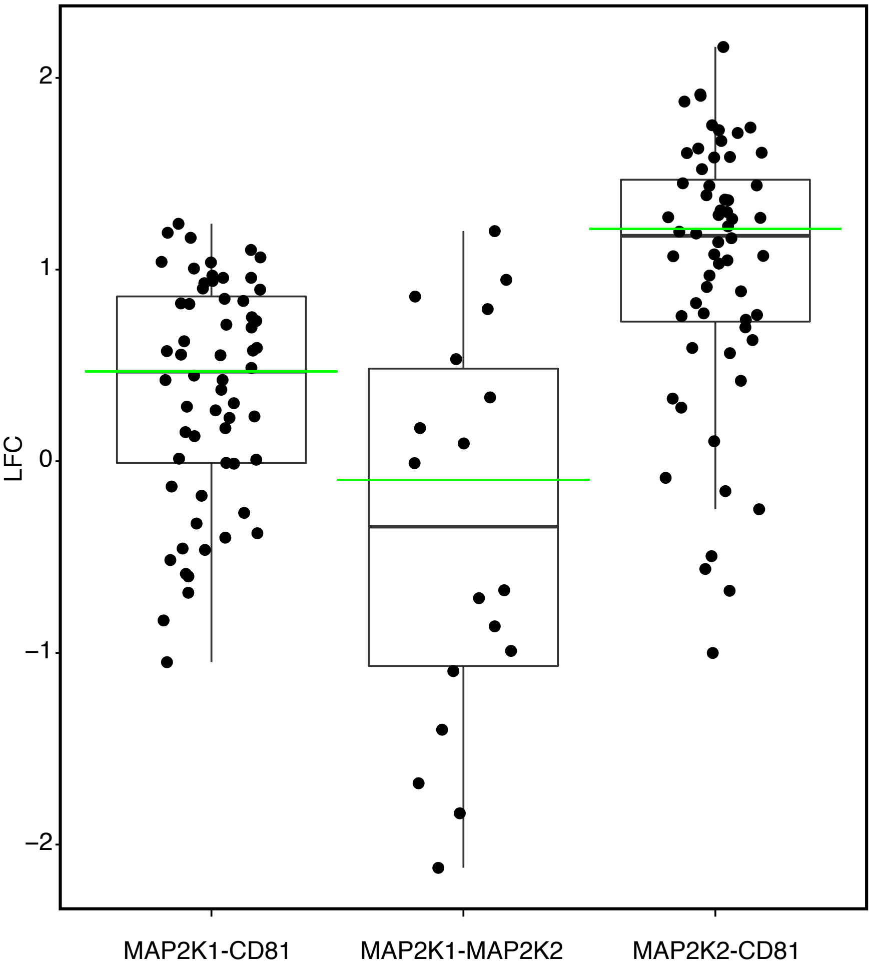

Figure S4: **Reagent variability in *MAP2K1-MAP2K2*.** Boxplots of LFCs corresponding to guide pairs that target *MAP2K1- CD81*, *MAP2K2-CD81*, and *MAP2K1- MAP2K2* in A549 in Big Papi. We observe a large guide variability in *MAP2K1-MAP2K2*, ranging from 1 (cell growth) to -2 (cell depletion). This nearly bimodal distribution indicates that half of the guide pairs targeting *MAP2K1-MAP2K2* shows no significant lethality compared to the individual knockout of *MAP2K1* and *MAP2K2*, while the other half suggests modest lethality. Additionally, the guides targeting the individual genes also show high variability. Because of such extreme variability in the individual and combination settings, GEMINI does not identify *MAP2K1-MAP2K2* as a strong interaction, although it does identify this as a modest interaction ("strong" interaction score $= 0.56$, placing this gene pair in the top 17% of scores).

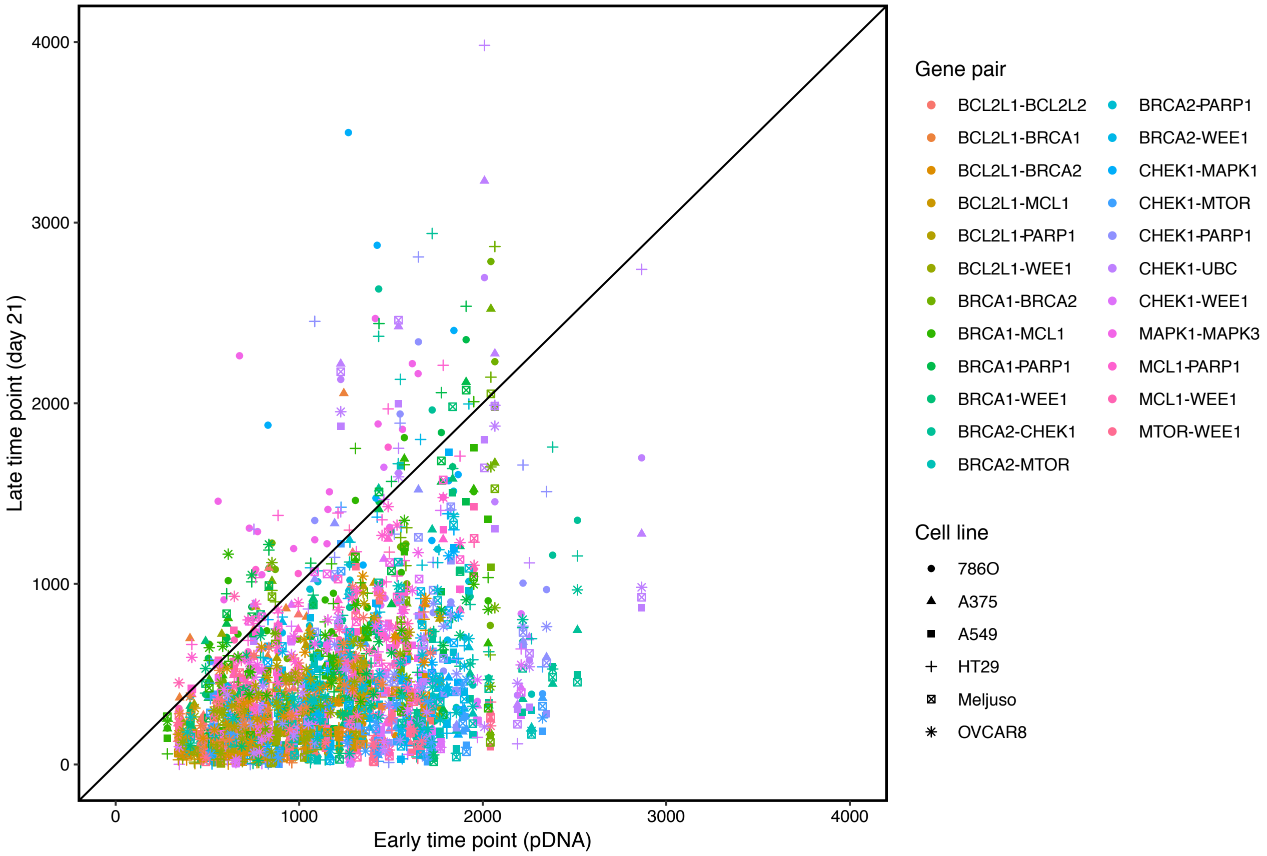

Figure S5: **Normalized read counts for the common interactions in Big Papi.** Similar to Figure S3 in Additional file 1, normalized counts were calculated for all cell lines screened in Big Papi. Common interactions found in 4 or more cell lines (Fig. 3a) are highlighted here, where colors indicate guide pairs targeting the same gene pair and shapes indicate different cell lines. The majority of guide pairs are represented more highly at the early time point, suggesting that common interactions show a dropout in counts across cell lines, consistent with GEMINI's findings. Note that all guide pairs have at least 169 or more raw counts at early time point.

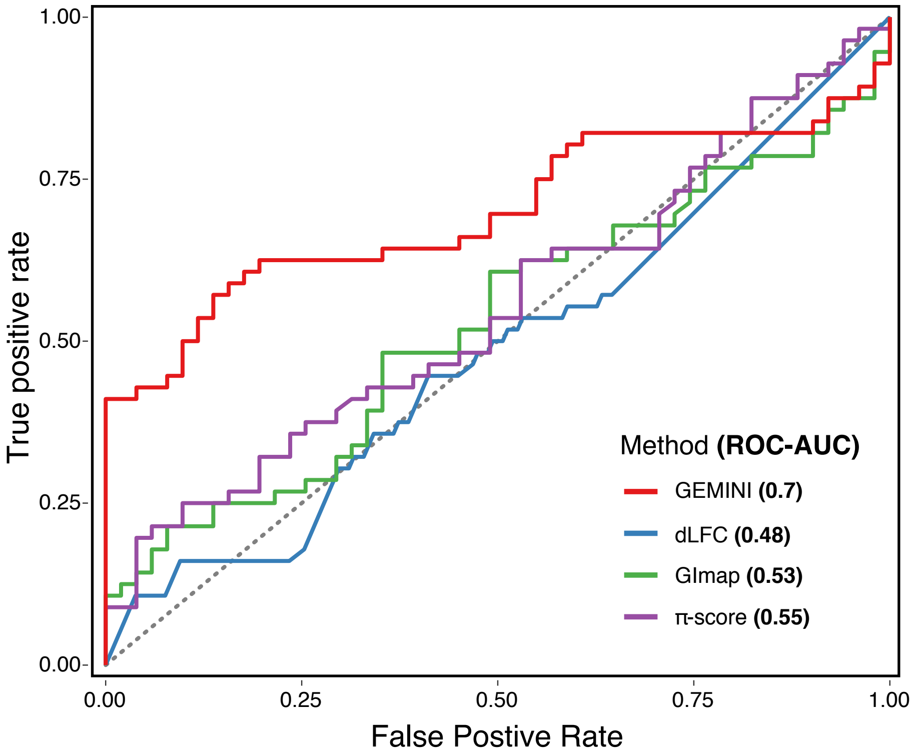

Figure S6: **ROC curves for GEMINI, π-score, GImap, and dLFC using Big Papi.** To enable a fair ROC comparison for these methods, we transformed GImap and GEMINI's "sensitive" scores to z-scores (by default, π-score generates z-score; GImap scores approximately follow a normal distribution). Note that GImap and π-score have negative z-scores for lethal interactions, while positive z-scores are lethal in GEMINI. For GImap and π-score, positive predictions were set to gene pairs with z-scores less than an increasing cut-off, and for GEMINI, positive predictions were set to gene pairs with the negative of the z-scores less than the increasing cut-off. For dLFC, gene pairs with FDRs less than cut-offs from 0 to 1 were treated as positive predictions. Here, we only considered lethal interactions (i.e. positive predictions) that were found in at least four cell lines of Big Papi dataset. ROC curves were then calculated according to the positive predictions and the defined true postive and true negative sets (Supplementary Information in Additional file 1), where GEMINI, π-score, GImap, and dLFC achive an ROC-AUC of 0.7, 0.55, 0.53, and 0.48, respectively.

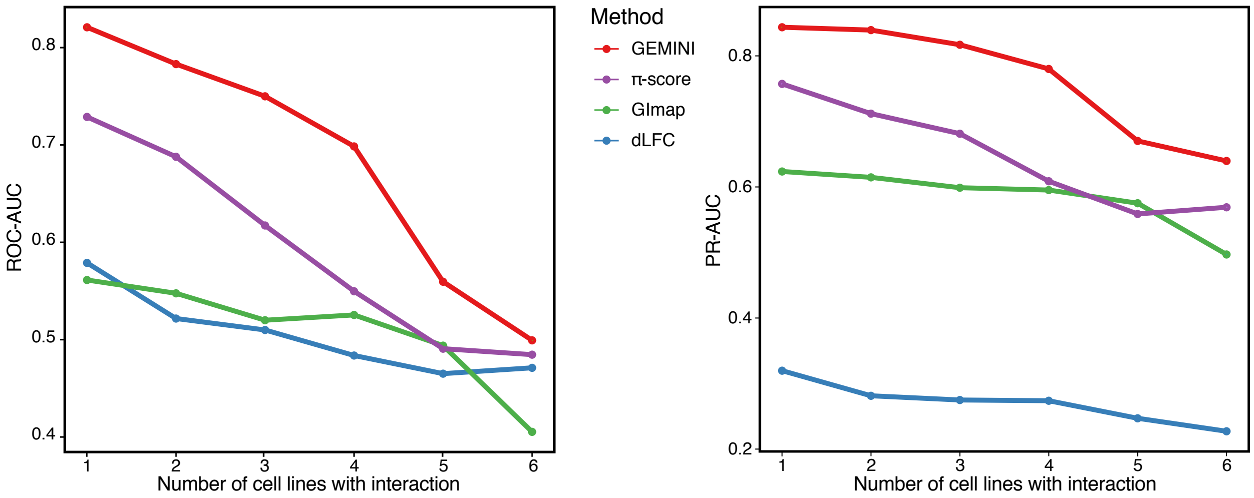

Figure S7: **Comparison of GEMINI to π-score, GImap, and dLFC for detection of lethal interactions across varying numbers of cell lines.** We applied all methods to the Big Papi dataset, and computed PR-AUC and ROC-AUC when lethal interactions found in at least a specific number of cell lines were treated as the positive prediction. AUC (y-axis) across different numbers of cell lines (x-axis) are shown for all methods. GEMINI consistently outperforms other methods, suggesting that GEMINI better identifies common interactions among samples.

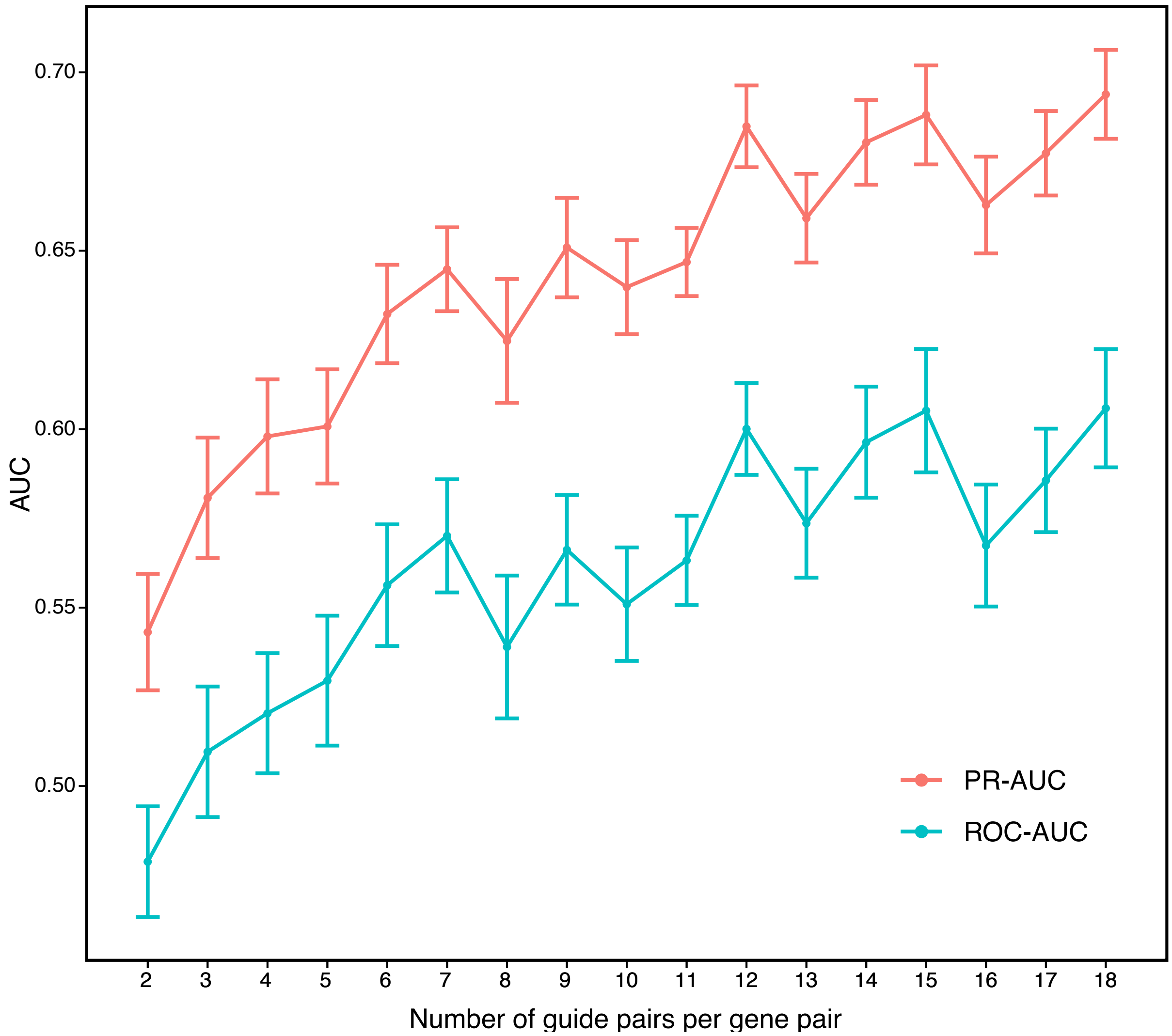

Figure S8: **GEMINI performance as a function of guide pairs per gene pair.** We randomly down-sampled from the Big Papi dataset, selecting a specific number of guide pairs per gene pair (from 2 to 18 pairs) that resulted in 340 datasets. We emphasize that i) the Big Papi dataset has 18 guide pairs for all gene pairs except for those including negative controls, and ii) our random subsets have higher variation in gene pairs compared to the full symmetric data set. GEMINI was applicable to all datasets (but did not achieve convergence in 20 iterations for %6 of datasets) while π-score and GImap were not applicable to any generated dataset. We calculated GEMINI's PR-AUC (red) and ROC-AUC (blue) across all datasets for lethal interactions found in at least four cell lines. In both cases, AUC (y-axis) improves as the number of guide pairs per gene pair increases (x-axis).

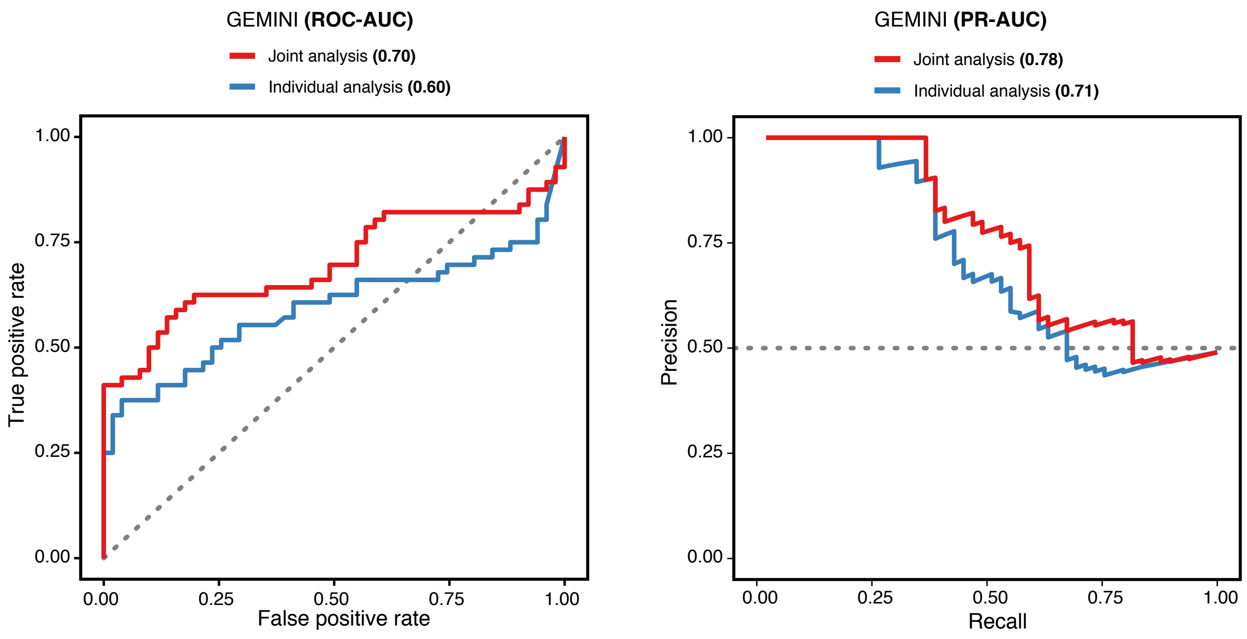

Figure S9: **Joint analysis of samples increases area under the precision-recall and receiver operator characteristic curves.** We applied GEMINI to each of Big Papi's cell lines individually, and calculated PR and ROC curves for lethal interactions found in at least four cell lines (blue). We similarly calculated these curves when GEMINI was applied to all cell lines simultaneously (red). We observe that our joint analysis of cell lines improves PR-AUC by 0.07 and ROC-AUC by 0.10, noting that this integrative analysis is not incorporated in any existing methods.

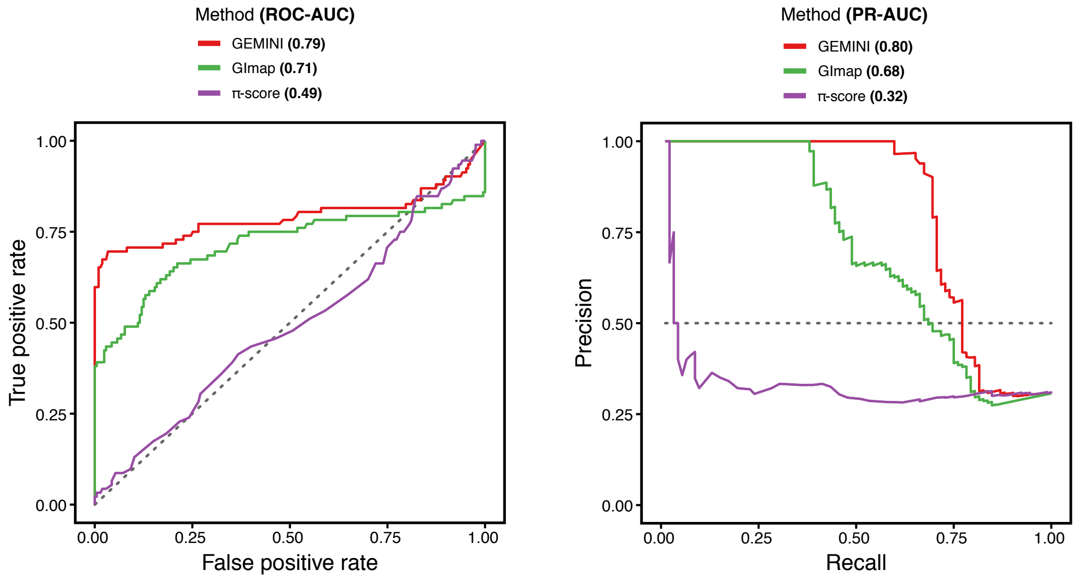

Figure S10: **ROC and PR curves for GEMINI, GImap, and π-score using CDKO.** We applied GEMINI, GImap, and π-score to the CDKO dataset. We could not run dLFC since its implementation is not available, and only its final results are reported for Big Papi. ROC and PR were calculated for all methods using a set of true positive interactions from SynLethDB and true negative interactions from half of the non-targeting negative control guides (named "safe" guides in CDKO) paired with all other genes. The other half of the "safe" guides were used as negative controls for all methods. The ROC and PR calculations were performed similarly to those in Fig. 3b and Figure S6 in Additional file 1, but only in one cell line (K562). GEMINI consistently outperforms other methods in a large library of $490,000$ guide pairs. Specifically, GEMINI, GImap, and π-score achieve ROC-AUCs of 0.79, 0.71, and 0.49, and PR-AUCs of 0.80, 0.68, and 0.32.

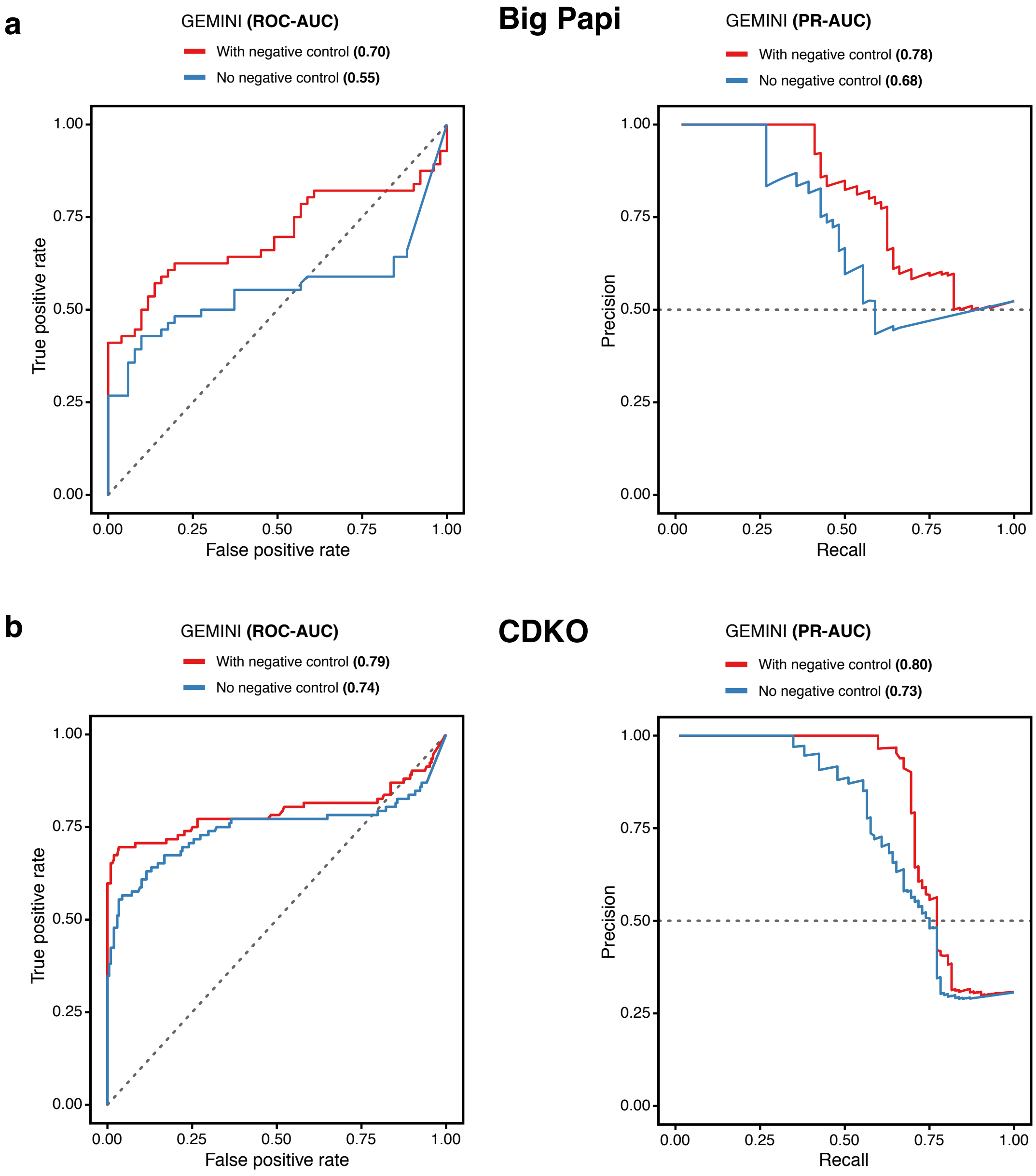

Figure S11: **CAVI initialization based on negative controls improves GEMINI performance.** (**a**) GEMINI was run on the Big Papi dataset without a specified negative control, and again when *CD81* was specified as a negative control. ROC and PR curves were calculated according to Fig. 3b and Figure S6 in Additional file 1. When a negative control is utilized in the initialization process, GEMINI performance improves by 0.15 in ROC-AUC, and 0.1 in PR-AUC. (**b**) GEMINI was run on the CDKO data set without a specified negative control, and again when half of the "safe" guides were used as negative controls. ROC and PR curves were calculated according to Figure S10 in Additional file 1. GEMINI performs robustly in both cases, but still shows an increase in performance when a negative control is provided.

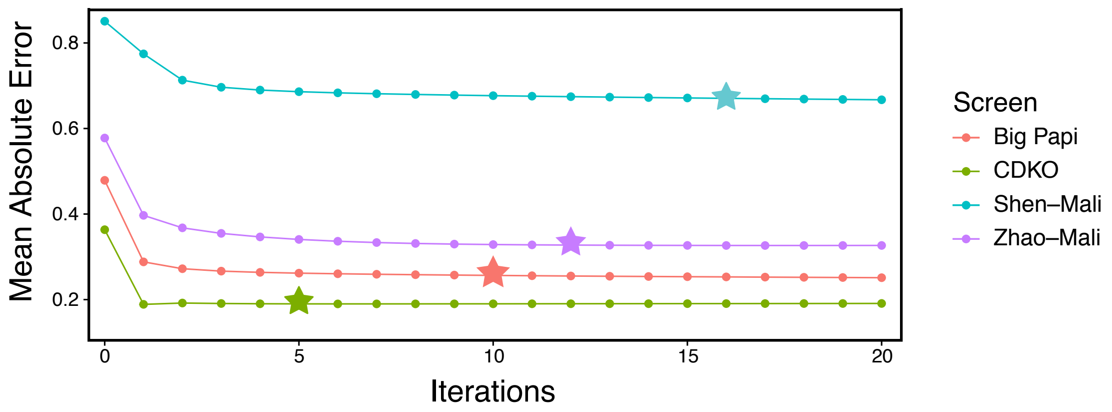

Figure S12: **GEMINI convergence rate.** The absolute difference between the observed and GEMINI's predicted LFCs were calculated for CDKO, Zhao-Mali, Shen-Mali, and Big Papi screens. The average of these values across all guide pairs and cell lines (mean absolute error) is shown at each iteration. Stars specify the iteration at which changes in the mean absolute error are less than 0.001 compared to the previous iteration (i.e. convergence achieved).

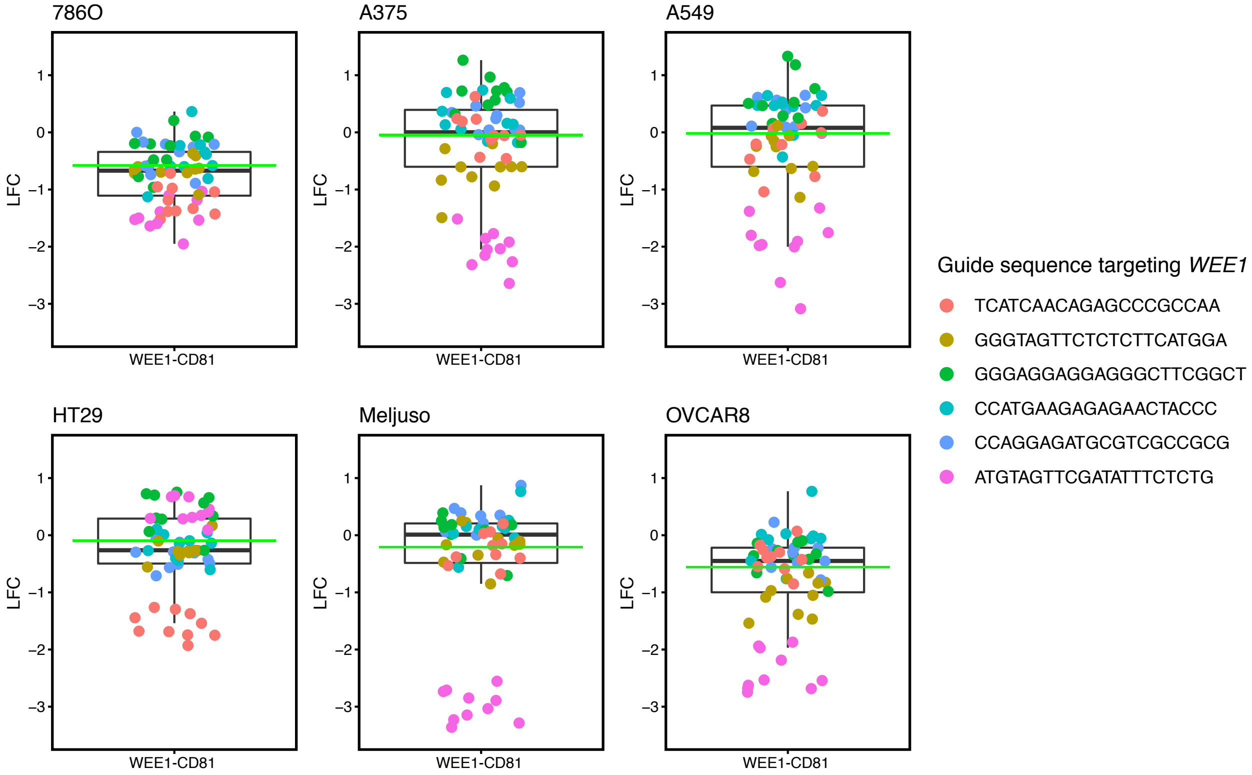

Figure S13: **Extreme variations in guide activity may cause an incorrect inference of individual gene effects.** LFCs of each guide targeting *WEE1* paired with guides targeting *CD81* (negative control) are depicted in the same color across all cell lines in Big Papi, with corresponding sequences in the legend. Although the majority of guides suggest no phenotype, at least one guide in each cell line displays a lethal phenotype that agrees with previously conducted single-knockout CRISPR screens [1]. Note that individual gene effects inferred by GEMINI with default priors (green lines) approximately follow the median LFC.

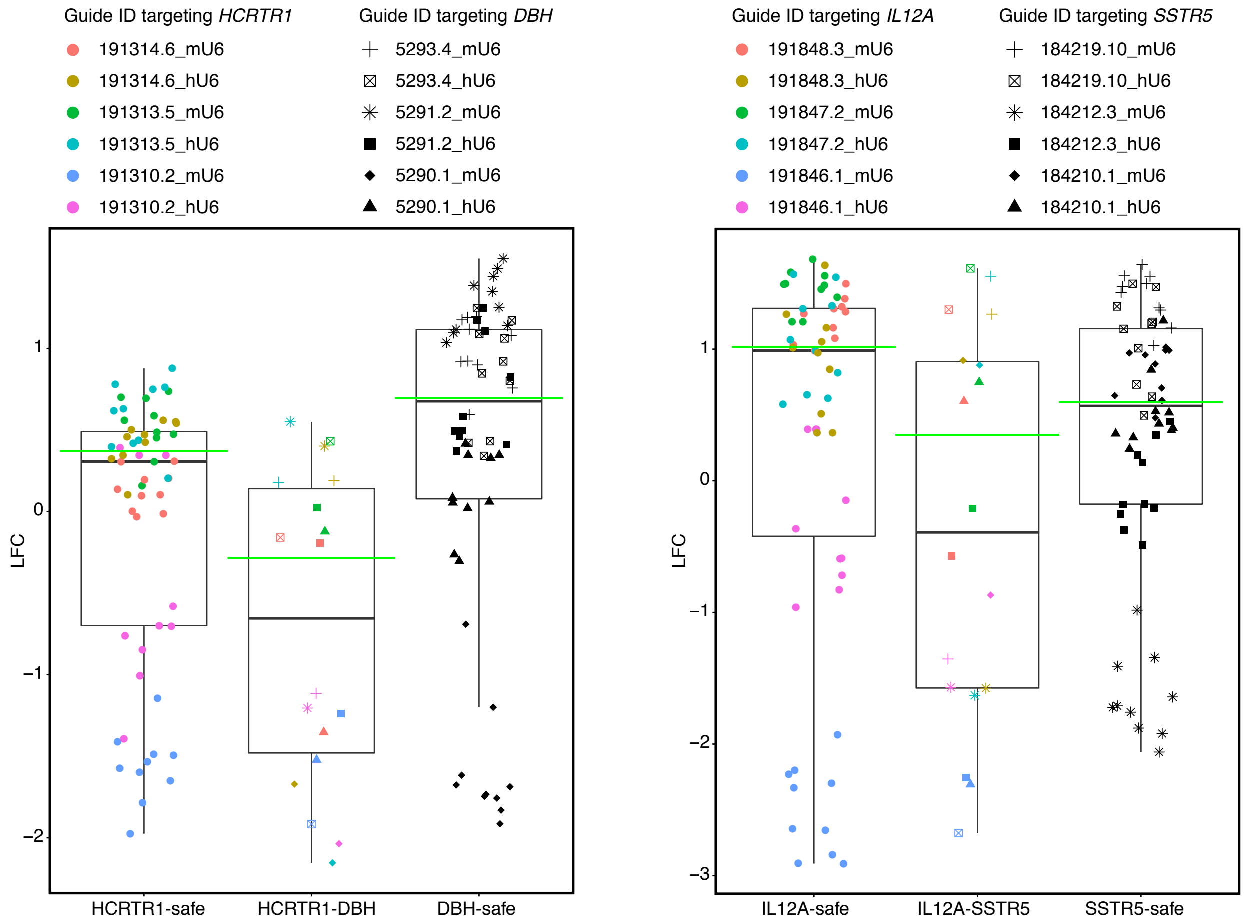

Figure S14: **Extreme variations in guide activity may cause an incorrect inference of combination gene effects.** Colors indicate unique guides targeting *HCRTR1* (similarly for *IL12A* on right), while shapes indicate unique guides targeting *DBH* (similarly for *SSTR5* on right) in the K562 cell line in CDKO. Guide pairs targeting both genes are marked by the corresponding colors and shapes. Here, the negative control is denoted by "safe", and guide pairs including "safe" follow the same color structure as described in Figure S13 in Additional file 1. Left: any guide that is paired with 191310.2_mU6, 191310.2_hU6, or 5290.1_mU6 shows a notable lethal phenotype, while other guide pairs on average show no phenotype. GEMINI, when applied to this bimodal data using default priors, results in the total gene combination effect that is somewhat less than both individual gene effects (green lines). Consequently, a weak "senstive" interaction score might be detected. If guide pairs with strong depletion exhibit the true signal, not only would there be no detected interaction, but GEMINI's individual and combination effects would also be incorrect. Right: we again observe an extreme variability in guide activity, leading to uncertainty in GEMINI's inferred values.

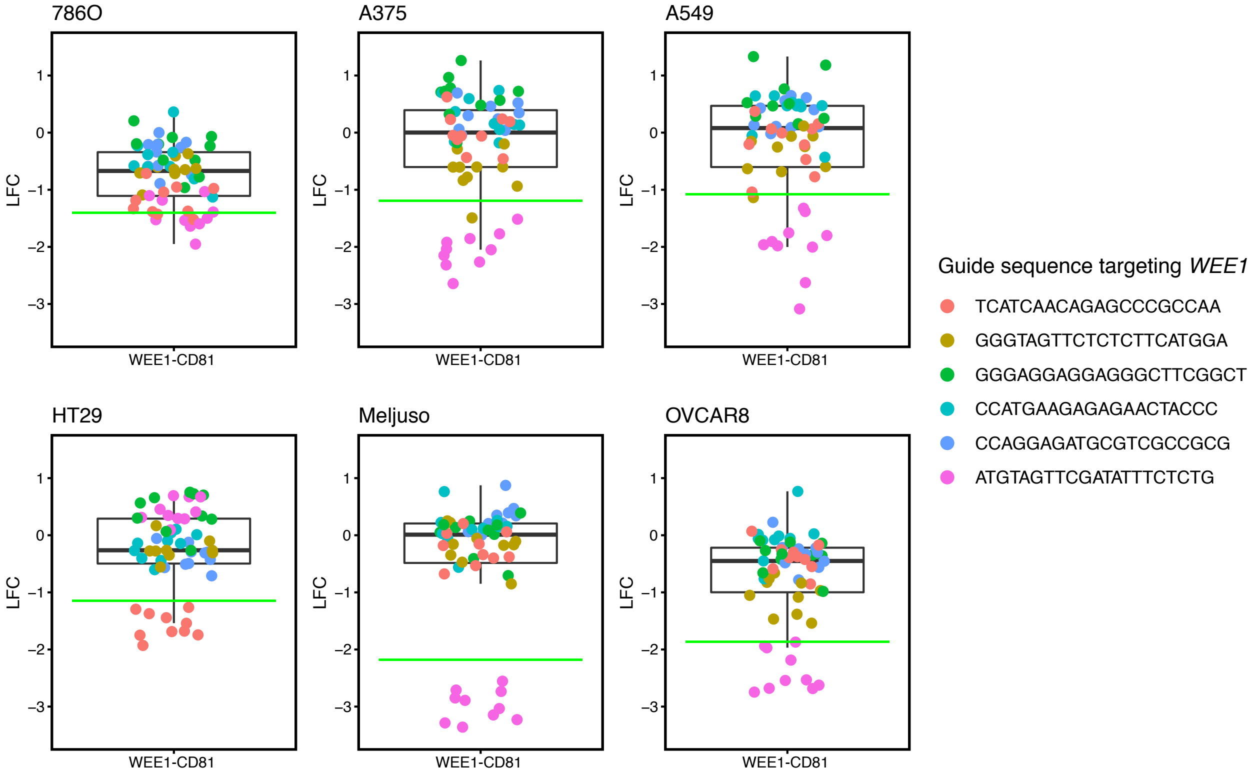

Figure S15: **Incorporation of guide activity into GEMINI for a better estimation of individual gene essentiality.** Individual gene effect, inferred by GEMINI with modified priors, for *WEE1* across all cell lines in Big Papi is shown. LFCs of guide pairs, guide sequences, and colors are consistent with Figure S13 in Additional file 1. Here, stronger confidence ($\mu_x = 1$, $\sigma_x = 1e-3$) was placed on guides exhibiting a strong lethal phenotype while weaker confidence ($\mu_x = 0.1$, $\sigma_x = 1e-3$) was placed on other guides. Consequently, GEMINI's inferred values are more biased towards guides with high confidence, and are consistent with what is observed in the single-gene knockout CRISPR screens [1].

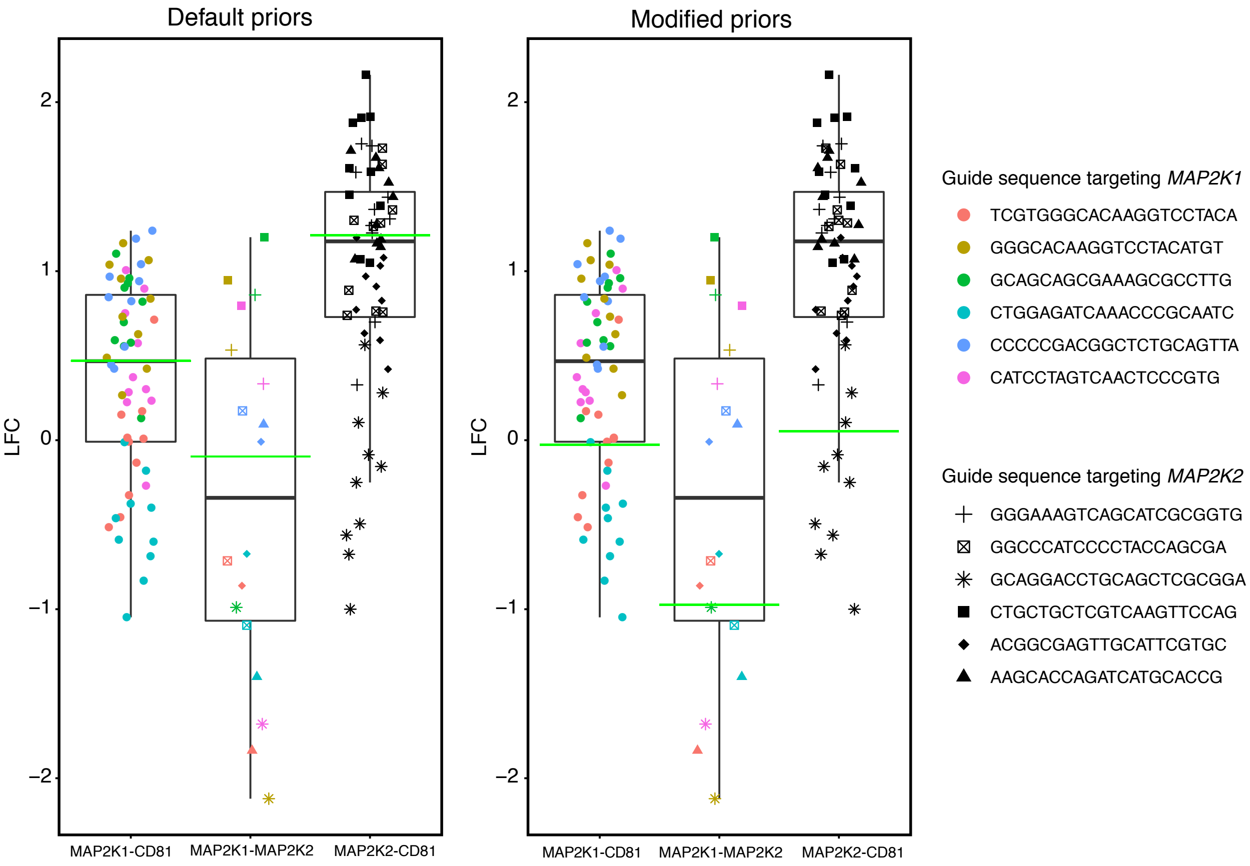

Figure S16: **Incorporation of guide activity into GEMINI for a better identification of true interactions.** Individual gene effects and total gene combination effect, inferred by GEMINI with default and modified priors, for *MAP2K1*-*MAP2K2* in the A549 cell line of Big Papi are shown. Guides are colored similar to Figure S14 in Additional file 1. Left: GEMINI's inference with default priors only captures a modest interaction. Right: GEMINI was run with adjusted priors, where any guide pairs including TCGTGGGCACAAGGTCCTACA and CTGGAGATCAAACCCGCAATC targeting *MAP2K1* and GCAGGACCTGCAGCTCGCGGA targeting *MAP2K2* were assigned a higher confidence ($\mu_x = 1$, $\sigma_x = 1e-3$) while other guides were assigned a lower confidence ($\mu_x = 0.1$, $\sigma_x = 1e-3$). Guides were assigned a high confidence based on their strong negative phenotypes when paired with the negative control. With modified priors, GEMINI identifies the well-characterized *MAP2K1*-*MAP2K2* as a strong interaction [2].

2 Supplementary Methods

2.1 Calculation of LFCs

Similar to the analysis of single knockout screens [3], we calculate the LFC of guide i targeting gene g (i.e. g_i) and guide j targeting gene h (i.e. h_j) in each sample. We first normalize the raw read counts for every replicate by its total number of counts, called read counts hereafter. We then compute the LFC for (g_i, h_j) pair at sample l according to

$$D_{g_i,h_j,l} = \frac{1}{N_l}\sum_{r=1}^{N_l} C_{g_i,h_j,l,r} - \frac{1}{N_p}\sum_{r=1}^{N_p} C_{g_i,h_j,p,r}$$

where N_l is the number of replicates for sample l and N_p is the number of replicates for pDNA (or early time point). Let $c_{g_i,h_j,l,r} = \log_2(\text{count}_{g_i,h_j,l,r} + \text{constant})$ be the log-transformation of the read count of (g_i, h_j) pair for the rth replicate in sample l. We introduce the constant value in the log-transformation (default is 32) to reduce noises from low read counts. We define $C_{g_i,h_j,l,r} = c_{g_i,h_j,l,r} - \text{median}(c_{:,:,l,r})$, with the median function obtained across all guides and all genes. Similarly, we define $C_{g_i,h_j,p,r} = c_{g_i,h_j,p,r} - \text{median}(c_{:,:,p,r})$ where $c_{g_i,h_j,p,r} = \log_2(\text{count}_{g_i,h_j,p,r} + \text{constant})$ and p represents pDNA.

2.2 Precision of LFCs

The precision of $D_{g_i,h_j,l}$ is modeled by $\tau_{g_i,h_j,l}$. We compute the prior parameters for $\tau_{g_i,h_j,l}$ as

$$\alpha_{g_i,h_j,l} = \kappa,$$
$$\beta_{g_i,h_j,l} = \kappa\left(\sigma^2_{g_i,h_j,p} + \sigma^2_{g_i,h_j,l}\right),$$

where κ determines the skewness of the prior (default is 0.5); $\sigma^2_{g_i,h_j,p}$ is the empirical variance of (g_i, h_j) pair, calculated using pDNA's replicates; and $\sigma^2_{g_i,h_j,l}$ is the empirical variance of (g_i, h_j) pair, calculated using sample l's replicates. The prior mean is $1/\left(\sigma^2_{g_i,h_j,p} + \sigma^2_{g_i,h_j,l}\right)$, and represents the expected precision of $D_{g_i,h_j,l}$.

As defaults, GEMINI uses the above empirical estimates for τ. If the smoothed emrical estimates are required by users, GEMINI applies the same approach in [3] by treating guide pairs as individual guides.

2.3 Coordinate ascent variational inference

We focus on a family of distributions where the latent variables are mutually independent, named the mean-field varitional family. Specifically, the posterior distribution of x, y, s, and τ is approximated by

$$q(x, y, s, \tau) = \prod_{g,i} q(x_{g_i}) \prod_{g,i,h,j} q(x_{g_i,h_j}) \prod_{g,l} q(y_{g,l}) \prod_{g,h,l} q(s_{g,h,l}) \prod_{g,i,h,j,l} q(\tau_{g_i,h_j,l}).$$

In variational inference setup, the optimal q minimizes the Kullback-Leibler (KL) divergence of q from the true posterior distribution. The optimal q for each latent variable, for instance x_{g_i}, is proportional to

$$\mathbb{E}_{-x_{g_i}}[\ln f(D, x, y, s, \tau)],$$

which is the expectation of log of the joint distribution over all variables except for x_{g_i}. We calculate the closed-form expressions of these expectations, and use them as coordinate updates in the coordinate ascent variational inference (CAVI) algorithm to acheive the optimal q. See [4, 5] for more details. The closed-form updates are

- $q(x_{g_i}) \sim \mathcal{N}(\mu_{x_{g_i}}, \sigma^2_{x_{g_i}})$ where

$$\mu_{x_{g_i}} = \frac{\frac{\mu_x}{\sigma_x^2} + \sum_{h,j,l} \mathbb{E}[y_{g,l}]\mathbb{E}[\tau_{g_i,h_j,l}]\Big(\mathbb{0}(h)(D_{g_i,h_j,l} - \mathbb{E}[x_{h_j}]\mathbb{E}[y_{h,l}] - \mathbb{E}[x_{g_i,h_j}]\mathbb{E}[s_{g,h,l}]) + \mathbb{1}(h)D_{g_i,h_j,l}\Big)}{\frac{1}{\sigma_x^2} + \sum_{h,j,l} \mathbb{E}[y^2_{gl}]\mathbb{E}[\tau_{g_i,h_j,l}]},$$

$$\sigma^2_{x_{g_i}} = \frac{1}{\frac{1}{\sigma_x^2} + \sum_{h,j,l} \mathbb{E}[y^2_{g,l}]\mathbb{E}[\tau_{g_i,h_j,l}]}.$$

Here, $\mathbb{0}(h)$ is one if h does not belong to the set of negative control genes, zero otherwise. The indicator function $\mathbb{1}(h)$ is one if h belongs to the set of negative control genes, zero otherwise.

- $q(x_{g_i,h_j}) \sim \mathcal{N}(\mu_{x_{g_i,h_j}}, \sigma^2_{x_{g_i,h_j}})$ where

$$\mu_{x_{g_i,h_j}} = \frac{\frac{\mu_x}{\sigma_x^2} + \sum_l \mathbb{E}[s_{g,h,l}]\mathbb{E}[\tau_{g_i,h_j,l}]\Big(D_{g_i,h_j,l} - \mathbb{E}[x_{g_j}]\mathbb{E}[y_{g,l}] - \mathbb{E}[x_{h_j}]\mathbb{E}[y_{h,l}]\Big)}{\frac{1}{\sigma_x^2} + \sum_l \mathbb{E}[s^2_{g,h,l}]\mathbb{E}[\tau_{g_i,h_j,l}]},$$

$$\sigma^2_{x_{g_i,h_j}} = \frac{1}{\frac{1}{\sigma_x^2} + \sum_l \mathbb{E}[s^2_{g,h,l}]\mathbb{E}[\tau_{g_i,h_j,l}]}.$$

- $q(y_{g,l}) \sim \mathcal{N}(\mu_{y_{g,l}}, \sigma^2_{y_{g,l}})$ where

$$\mu_{y_{g,l}} = \frac{\frac{\mu_y}{\sigma_y^2} + \sum_{h,i,j} \mathbb{E}[x_{g_i}]\mathbb{E}[\tau_{g_i,h_j,l}]\Big(\mathbb{0}(h)(D_{g_i,h_j,l} - \mathbb{E}[x_{h_j}]\mathbb{E}[y_{h,l}] - \mathbb{E}[x_{g_i,h_j}]\mathbb{E}[s_{g,h,l}]) + \mathbb{1}(h)D_{g_i,h_j,l}\Big)}{\frac{1}{\sigma_y^2} + \sum_{h,i,j} \mathbb{E}[x^2_{g_i}]\mathbb{E}[\tau_{g_i,h_j,l}]},$$

$$\sigma^2_{y_{g,l}} = \frac{1}{\frac{1}{\sigma_y^2} + \sum_{h,i,j} \mathbb{E}[x^2_{g_i}]\mathbb{E}[\tau_{g_i,h_j,l}]}.$$

- $q(s_{g,h,l}) \sim \mathcal{N}(\mu_{s_{g,h,l}}, \sigma^2_{s_{g,h,l}})$ where

$$\mu_{s_{g,h,l}} = \frac{\frac{\mu_s}{\sigma_s^2} + \sum_{i,j} \mathbb{E}[x_{g_i,h_j}]\mathbb{E}[\tau_{g_i,h_j,l}]\Big(D_{g_i,h_j,l} - \mathbb{E}[x_{g_j}]\mathbb{E}[y_{g,l}] - \mathbb{E}[x_{h_j}]\mathbb{E}[y_{h,l}]\Big)}{\frac{1}{\sigma_s^2} + \sum_{i,j} \mathbb{E}[x^2_{g_i,h_j}]\mathbb{E}[\tau_{g_i,h_j,l}]},$$

$$\sigma^2_{s_{g,h,l}} = \frac{1}{\frac{1}{\sigma_s^2} + \sum_{i,j} \mathbb{E}[x^2_{g_i,h_j}]\mathbb{E}[\tau_{g_i,h_j,l}]}.$$

- $q(\tau_{g_i,h_j,l}) \sim \Gamma(\alpha_{\tau_{g_i,h_j,l}}, \beta_{\tau_{g_i,h_j,l}})$ where

$$\alpha_{\tau_{g_i,h_j,l}} = \alpha_{g_i,h_j,l} + 0.5,$$
$$\beta_{\tau_{g_i,h_j,l}} = \beta_{g_i,h_j,l} + 0.5\Big(\mathbb{0}(g)\mathbb{0}(h)A + \mathbb{1}(g)\mathbb{0}(h)B + \mathbb{0}(g)\mathbb{1}(h)C + \mathbb{1}(g)\mathbb{1}(h)D^2_{g_i,h_j,l}\Big),$$
$$\mathbb{E}(\tau_{g_i,h_j,l}) = \frac{\alpha_{\tau_{g_i,h_j,l}}}{\beta_{\tau_{g_i,h_j,l}}}.$$

The prior parameters $\alpha_{g_i,h_j,l}$ and $\beta_{g_i,h_j,l}$ are empirically determined (see the previous subsection), and

$$\begin{aligned}A =&\mathbb{E}[D_{g_i,h_j,l} - x_{g_i}y_{g,l} - x_{h_j}y_{h,l} - x_{g_i,h_j}s_{g,h,l}]^2\\=&D^2_{g_i,h_j,l} - 2D_{g_i,h_j,l}\Big(\mathbb{E}[x_{g_i}]\mathbb{E}[y_{g,l}] + \mathbb{E}[x_{h_j}]\mathbb{E}[y_{h,l}] + \mathbb{E}[x_{g_i,h_j}]\mathbb{E}[s_{g,h,l}]\Big) + \mathbb{E}[x^2_{g_i}]\mathbb{E}[y^2_{g,l}]+\\&2\mathbb{E}[x_{g_i}]\mathbb{E}[y_{g,l}]\Big(\mathbb{E}[x_{h_j}]\mathbb{E}[y_{h,l}] + \mathbb{E}[x_{g_i,h_j}]\mathbb{E}[s_{g,h,l}]\Big) + \mathbb{E}[x^2_{h_j}]\mathbb{E}[y^2_{h,l}] + \mathbb{E}[x^2_{g_i,h_j}]\mathbb{E}[s^2_{g,h,l}]+\\&2\mathbb{E}[x_{h_j}]\mathbb{E}[y_{h,l}]\mathbb{E}[x_{g_i,h_j}]\mathbb{E}[s_{g,h,l}],\end{aligned}$$

$$\begin{aligned}B =&\mathbb{E}[D_{g_i,h_j,l} - x_{h_j}y_{h,l}]^2\\=&D^2_{g_i,h_j,l} - 2D_{g_i,h_j,l}\mathbb{E}[x_{h_j}]\mathbb{E}[y_{h,l}] + \mathbb{E}[x^2_{h_j}]\mathbb{E}[y^2_{h,l}],\end{aligned}$$

$$\begin{aligned}C =&\mathbb{E}[D_{g_i,h_j,l} - x_{g_j}y_{g,l}]^2\\=&D^2_{g_i,h_j,l} - 2D_{g_i,h_j,l}\mathbb{E}[x_{g_j}]\mathbb{E}[y_{g,l}] + \mathbb{E}[x^2_{g_j}]\mathbb{E}[y^2_{g,l}].\end{aligned}$$

In the above updates, we assume that x_{g_i} is one if g is a negative control; $x_{g_ih_j}$ is one if g or h is a negative control; $y_{g,l}$ is zero if g is a negative control; and $s_{g,h,l}$ is zero if g or h is a negative control.

We essentially implement the CAVI algorithm as follows.

1. Initialize the latent variables according to

$$\begin{aligned}&\mathbb{E}[x_{g_i}] = 1, \quad \mathbb{E}[x^2_{g_i}] = 1, \quad \mathbb{E}[x_{g_i,h_j}] = 1, \quad \mathbb{E}[x^2_{g_i,h_j}] = 1,\\&\mathbb{E}[y_{g,l}] = \text{median}(D_{g_i,h_j,l}) \text{ across all } i \text{ and } h_j \text{ with } \mathbb{1}(h) = 1, \quad \mathbb{E}[y^2_{g,l}] = \mathbb{E}[y_{g,l}]^2,\\&\mathbb{E}[s_{g,h,l}] = \text{median}(D_{g_i,h_j,l}) - \mathbb{E}[y_{g,l}] - \mathbb{E}[y_{h,l}] \text{ where the mdian function is computed across all } i \text{ and } j,\\&\mathbb{E}[s^2_{g,h,l}] = \mathbb{E}[s_{g,h,l}]^2, \quad \mathbb{E}(\tau_{g_i,h_j,l}) = \frac{\alpha_{g_i,h_j,l}}{\beta_{g_i,h_j,l}}.\end{aligned}$$

2. Update each latent variable using the closed-from equations, while holding the other variables fixed.

3. Calculate the following mean absolute error (MAE) after updating all variables (i.e. one iteration). Convergence is achieved if the change in MAE is less than .001 compared to the previous iteration.

$$\text{MAE} = \text{mean}\Big(\Big|D_{g_i,h_j,l} - \mathbb{E}[x_{g_i}]\mathbb{E}[y_{g,l}] - \mathbb{E}[x_{h_j}]\mathbb{E}[y_{h,l}] - \mathbb{E}[x_{g_i,h_j}]\mathbb{E}[s_{g,h,l}]\Big|\Big) \text{ across all } g_i,\ h_j \text{ and } l.$$

We emphasize that CAVI only guarantees convergence to a local optimum, and several random initializations are often needed to obtain a solution close to the true posterior. However, it can be computationally expensive to repeat CAVI with different initializations, especially for high-dimensional datasets. We instead utilize negative control genes to initialize variables, which resulted in the highest PR-AUC and ROC-AUC (shown in Fig. 3b and Figures S6 and S10 in Additional file 1) compared to random initializations.

2.4 FDR calculation

Given a set of known non-interacting gene pairs, we fit a mixture of normal distributions to GEMINI scores of the non-interacting set (i.e. null distribution). We apply the expectation–maximization algorithm to estimate the mixture distribution ("mixtools" package in R). Next, for each gene pair in the combinatorial screen, we calculate the right tail probability that the null distribution generates a GEMINI score greater than the GEMINI score of that gene pair (p-values). We also adjust these p-values using Benjamini-Hochberg's method [6].

For the Big Papi dataset, we used gene pairs that include one of the negative controls, *HPRT intron* or *6T*, as the non-interacting set, while using the remaining negative control, *CD81*, to initialize gene and combination effects in

GEMINI's CAVI algorithm. This non-interacting set might result in an overestimation of the number of significant hits due to the construction of null distribution solely based on negative controls. However, GEMINI still outputs scores that capture the relative strengths of interactions in each sample. For other screens, we had an insufficient number of non-interacting pairs to define a reliable null distribution for the calculation of FDR, but GEMINI scores are reported in Table S1 in Additional file 2. We emphasize that in the method comparison section, we did not use FDRs because the true negative set was identical to the non-interacting set. Instead, we transformed GEMINI scores to z-scores to enable a fair comparison.

3 Supplementary Information

3.1 Identification of recovery interactions

We define recovery interactions where a single gene perturbation results in a loss of viability, and the perturbation of a second gene partially or completely rescues the loss of viability [7]. This differs from the definition of a buffering interaction [8], where the perturbation of two genes simultaneously results in a total effect that is less than the addition of effects from perturbing each gene. Using our definition, we applied GEMINI to all publicly available combinatorial CRISPR knockout screens and identified interactions with patterns of recovery (Table S1 in Additional file 2).

A systematic assessment of all recovery interactions is not possible, as these interactions have not yet been well-characterized. However, the Big Papi Apoptotic library presents a unique case for evaluating our approach. This library includes all BCL2-family proteins involved in anti-apoptotic and pro-apoptotic function, in an all-by-all format, as well as the same controls used in the Big Papi SynLet library. Cells screened with the Apoptotic library were also subjected to different drug treatments, enabling the discovery of context-specific interactions. From these screens, we identified *BAX-MCL1* as a strong recovery interaction (Figure S1 in Additional file 1). *MCL1* is reported to prevent BAX and BAK oligomerization [9], thereby preventing mitochondrial outer membrane permeabilization (MOMP) and cytochrome *c* release, thus negatively regulating apoptotic cell death. In the context of commonly used BH3-mimetics, such as ABT-263 and A-1331852, other functionally redundant members of the anti-apoptotic family are inhibited such as BCL2, BCL2L1, and BCL2L2, while MCL1 is not completely inhibited from its anti-apoptotic function [10, 2]. Here, the knockout of *MCL1* results in a significant drop in viability as cells treated with BH3-mimetics are particularly sensitive to the anti-apoptotic activity of *MCL1*. Knockout of *BAX* prevents MOMP-mediated apoptosis, and thus simultaneous knockout of *MCL1* and *BAX* rescues cell viability. This example demonstrates GEMINI's ability to characterize biologically relevant recovery interactions in combinatorial screens that were not otherwise detectable by existing methods.

3.2 Constructing true positive and true negative interaction sets

We used data presented by Srivas *et al.* [11] to construct a validation set for Big Papi. This data was generated from a screen to assess synthetic lethality in humans. Genetic interactions were assessed in HeLa cells by pairing drugs and shRNAs targeting yeast orthologs that were found to interact in *S. cerevisiae*. Because the screen was performed using RNA interference, a system with reportedly high off-target activity [12], we recognize that the genetic interactions identified by Srivas *et al.* may not reflect the absolute truth. However, in the absence of systematic arrayed validation, we used the strongest conserved interactions identified by Srivas *et al.* (p-value < 0.001) to construct our true positive set. Overall, we found 20 interactions that were also assessed in the Big Papi screen. In addition, similar to the approach taken in Najm *et al.* [13], we used gene family labels to classify 46 in-group interactions as true positives. Note that 10 interactions were in common between the in-group interactions and interactions from Srivas *et al.*, resulting in a total of 56 true positive interactions. Also note that the interactions unique to Srivas *et al.* represent out-of-group relationships that were considered to be negative by Najm *et al.*, which may have resulted in an underestimation of the true positive set. For our true negative set, we relied on pairs involving a negative control (*HPRT intron* or *6T*) that should not display any genetic interaction, leading to a set of 51 true negatives. The true positive and negative sets used for CDKO are described in the Results section in the manuscript.

3.3 Method comparison

We acquired implementations for GImap from `https://github.com/mhorlbeck/GImap_tools` and π-score from `https://github.com/ucsd-ccbb/mali-dual-crispr-pipeline`. To obtain gene-level results for π-score, we ran π-score for each sample using early and late time point. The obtained z-scores were averaged for each gene pair (given genes A and B, A-B and B-A were averaged to a single value). For GImap, given that doubling time for all cell lines was set to 10, gene-level interaction scores were calculated by averaging all guide-level GImap scores, and then again averaged across replicates to obtain a sample-level gene interaction score. GImap scores were also summarized such that any interaction scores corresponding to the same gene pair were averaged (given genes A and B, A-B and B-A were averaged to a single value). For dLFC, we used the results provided in Najm *et al.*. However, we could not run dLFC on CDKO since its implementation is not available.

To compare the performance of GEMINI and other methods, we first standardized the outputs of each method that show the strength of lethal interactions. As π-score already provides a z-score and GImap's results are roughly normally distributed, we computed z-scores for GImap. We also transformed GEMINI scores to a z-score, noting that GEMINI scores follow a mixture of distributions, which is not accurately reflected by z-score. Thus, z-score cutoffs were better suited for GImap and π-score, while GEMINI may have under-performed with z-score thresholds. Because the results from Najm *et al.* were reported as FDRs, z-scores were not computed, and instead FDR was used directly for dLFC.

The strongest lethal interaction has the lowest z-score for GImap and π-score, the highest z-score for GEMINI, and the smallest FDR for dLFC. To compare how well these methods identify lethal interaction, we used their scores and the true positive and true negative interaction sets to calculate PR and ROC curves. See Fig. 3b, Figures S6 and S10 in Additional file 1, and the manuscript for detailed results.

3.4 Reagent variability

Extreme reagent variability might result in inaccurate estimates of individual gene effects. For instance, in Big Papi, only two out of six guides that target *WEE1* show significant lethal phenotypes (LFC < -1) across all cell lines (Figure S13 in Additional file 1). Noting that *WEE1* was characterized as an essential gene [1], this clear bi-modality could be an indication of active or inactive guides. On the other hand, the majority of guides suggest no phenotype, and thus it is difficult for any unbiased algorithm to decide which mode would represent the true signal.

Explaining extreme reagent variability becomes more complex when inferring combination effects. To clarify this difficulty, consider the case where two essential genes are each targeted by 3 guides, and only one guide per gene shows a notable lethal phenotype. Assume that i) this gene pair does not synergize, and ii) the majority of single guides and guide pairs reflect the individual and combination effects, respectively. Consequently, single guides suggest no essentiality for individual genes, while guide pairs can suggest a lethal phenotype since "lethal" + "not lethal" are likely to result in "lethal". Hence, we mistakenly estimate a large combination effect compared to individual genes and predict a lethal interaction (Figure S14 in Additional file 1). This motivates a need for better understanding of variability in guide activity which could be subsequently incorporated in computational analysis to improve performance.

To account for this variation in GEMINI, inactive guides should be characterized prior to the analysis of a combinatorial screen. This information is then used as a prior in GEMINI to downweight guides with low activity or upweight guides with high activity. For example, imposing a stronger confidence on the two guides of *WEE1* that exhibit the highest activity, GEMINI estimates a notable lethal phenotype for this gene (Figure S15 in Additional file 1). If similar consideration is applied to guides targeting *MAP2K1-MAP2K2* in Big Papi, GEMINI also identifies this pair as a strong lethal interaction in *RAS*/*RAF* mutated lines (Figure S16 in Additional file 1), as was found in Najm *et al.*.

While an adjustment of the priors can improve GEMINI's inference, we emphasize that a comprehensive analysis of reagent variability is required to arrive at appropriate prior beliefs. Existing methods to create consistent on-target guides with minimal off-target effects have reduced variability in single-gene knockout screens [14, 15, 16], and primarily focused on sequence-related features, such as the biochemical properties of guide sequences and the existence of specific nucleotide subsequences. However, combinatorial guide design remains an area of open research, and other features may play a role in combination screens, including recombination rate, proximity of targeted loci, and the downstream functional impact in the protein coding sequence.

References

[1] Meyers, R. M. *et al.* Computational correction of copy number effect improves specificity of CRISPR–Cas9 essentiality screens in cancer cells. *Nature Genetics* **49**, 1779–1784 (2017).

[2] Aoidi, R., Maltais, A. & Charron, J. Functional redundancy of the kinases MEK1 and MEK2: Rescue of the Mek1 mutant phenotype by Mek2 knock-in reveals a protein threshold effect. *Sci. Signal.* **9**, ra9 (2016).

[3] Allen, F. *et al.* JACKS: joint analysis of CRISPR/Cas9 knockout screens. *Genome research* **29**, 464–471 (2019).

[4] Bishop, C. M. *Pattern Recognition and Machine Learning (Information Science and Statistics)* (Springer-Verlag, Berlin, Heidelberg, 2006).

[5] Blei, D. M., Kucukelbir, A. & McAuliffe, J. D. Variational inference: A review for statisticians. *Journal of the American Statistical Association* **112**, 859–877 (2017).

[6] Benjamini, Y. & Hochberg, Y. Controlling the false discovery rate: a practical and powerful approach to multiple testing. *Journal of the Royal Statistical Society. Series B (Methodological)* **57**, 289–300 (1995).

[7] Motter, A. E., Gulbahce, N., Almaas, E. & Barabási, A.-L. Predicting synthetic rescues in metabolic networks. *Molecular Systems Biology* **4**, 168 (2008).

[8] Hartman, J. L., Garvik, B. & Hartwell, L. Principles for the buffering of genetic variation. *Science* **291**, 1001–1004 (2001).

[9] Germain, M., Milburn, J. & Duronio, V. MCL-1 inhibits BAX in the absence of MCL-1/BAX interaction. *Journal of Biological Chemistry* **283**, 6384–6392 (2008).

[10] Oltersdorf, T. *et al.* An inhibitor of Bcl-2 family proteins induces regression of solid tumours. *Nature* **435**, 677–681 (2005).

[11] Srivas, R. *et al.* A network of conserved synthetic lethal interactions for exploration of precision cancer therapy. *Molecular Cell* **63**, 514–525 (2016).

[12] Scacheri, P. C. *et al.* Short interfering RNAs can induce unexpected and divergent changes in the levels of untargeted proteins in mammalian cells. *Proceedings of the National Academy of Sciences* **101**, 1892–1897 (2004).

[13] Najm, F. J. *et al.* Orthologous CRISPR–Cas9 enzymes for combinatorial genetic screens. *Nature Biotechnology* **36**, 179–189 (2018).

[14] Doench, J. G. *et al.* Optimized sgRNA design to maximize activity and minimize off-target effects of CRISPR-Cas9. *Nature biotechnology* **34**, 184–191 (2016).

[15] Chuai, G. *et al.* Deepcrispr: optimized crispr guide rna design by deep learning. *Genome biology* **19**, 80 (2018).

[16] Doench, J. G. *et al.* Rational design of highly active sgRNAs for CRISPR-Cas9–mediated gene inactivation. *Nature biotechnology* **32**, 1262–1267 (2014).
