# Supplementary material for: GEMINI: a variational Bayesian approach to identify genetic interactions from combinatorial CRISPR screens
Source: Genome Biol. 2019 Jul 12;20:137. doi: 10.1186/s13059-019-1745-9 (PMC6624979; doi:10.1186/s13059-019-1745-9)
Supplement: Supplementary file 3 — Review history. (DOCX 17 kb) [file 13059_2019_1745_MOESM3_ESM.docx]

Review History

**Comments to author and authors’ point-by-point responses to the reviewers’ comments:**

**Reviewer 1**

1. The manuscript would be better placed as a “Method” paper rather than a “Research” paper because the manuscript is focused on the development of the GEMINI methods; no new data or biological insights are presented/developed.

*We agree with your characterization of our manuscript, and have resubmitted it as a “Method” paper in Genome Biology.*

2. The Title of the manuscript could be changed to better reflect the emphasis on the new method. Thank you for this suggestion.

*We have modified the title to place higher emphasis on our new method.*

3. The description of the “strong” and “sensitive” interaction scores should be better explained in the text to improve readability and accessibility for non-computational readers. How are these calculated in layman’s terms and what do they represented? What thresholds are used?

*To clarify this distinction, we have improved the descriptions of these scoring systems both in the “Results” and “Methods” sections of the manuscript. To explain further, the “strong” score represents genetic interactions with high synergy, where the combination effect of an interaction is much greater than either of the individual effects. This can also be thought of as interactions with a phenotype that is “much more than additive”. On the other hand, the sensitive score best captures interactions where the phenotype of the pair is more lethal or more advantageous than either gene individually. To set the threshold for identifying significant interactions, we define false discovery rate using a given set of non-interacting genes and set the significance threshold to FDR < 0.01 (see Supplementary Methods in Additional file 1). If no such set is provided, GEMINI still outputs scores for both lethality and recovery, allowing the user to compare the relative strengths of interactions in each sample. We have also clarified this point in the manuscript.*

4. Have the authors considered non-specific CRISPR-Cas9 loss of fitness effects associated with copy number variation and structural alterations (Meyers et al. 2017 Nature Genetics; Goncalves et al. 2019 Genome Biology)? Could these lead to false positive genetic interactions in combinations screens and does GEMINI control/correct for this?

*We agree that the assessment of copy number variations will play an important role in dual CRISPR screens. Unfortunately, the published datasets are limited both by the number of genes and cell lines with copy number alterations, and therefore it is not possible to systematically evaluate any copy number effect in combinatorial screen. In anticipation of larger datasets, we are developing a pre-processing method, akin to those developed for single-gene knockout screens (de Weck et al. 2018 PLoS Computational Biology; Iorio et al. 2018 BMC Genomics). GEMINI will be able to handle data processed in such a fashion. This point has been clarified in the “Discussion” section.*

**Reviewer 2**

1. My biggest concern is that GEMINI is mainly tested on “Big Papi”’s dataset, already published by the same institution, with limited convincing evidence. This dataset is small scale (only 25 genes), small sample (6 cell lines). The results only pick up a few known synthetic lethal partners, and has no validation on the novel gene pairs they found. Furthermore, looking into more details of the results, the known ones appear to be problematic as well; for example, strong interactions between BRCA family and PARP1 is reported (page 3, line 61-62), but their p-values are not significant at all (Fig. 2b). This raises a question whether the method, or the dataset that this manuscript mainly relies on, is able to identify hits that are reliable.

*We agree that the Big Papi dataset covers a limited number of genes, but at the same time provides the greatest number of cell lines screened with a combinatorial CRISPR library. In addition, Big Papi contains several gene pairs that were identified as true positive synthetic lethal interactions in an orthogonal screen using drug and shRNA combinations by Srivas et al. Molecular Cell, 2016. This provided us with a set of unbiased and experimental cross-reference that we used to evaluate GEMINI in this dataset.*

*First, regarding the issue of statistical significance of the GEMINI’s hits in Fig. 2b, GEMINI did in fact, identify strongly significant interactions between BRCA family and PARP1 (FDR = 8.129 × 10−6 and GEMINI score = 1.320 for BRCA2 -PARP1 and FDR = 3.083 × 10−5 and GEMINI score = 1.050 for BRCA1 -PARP1 ). We apologize for any confusion that may have led the reviewer to interpret the results from the dLFC method shown in the lower section of Fig. 2b as those derived from GEMINI (GEMINI scores are in the upper left triangle). dLFC does not find any interaction between BRCA family members and PARP1 while GEMINI finds these interactions highly significant. We have clarified this in the caption of Fig. 2 and further improved Fig. 2 to clarify the labeling of the two analyses.*

*To address the major concern regarding the use of single validation dataset, we have now assessed GEMINI on CDKO, a combinatorial CRISPR knockout screen testing 207 genes in an all-by-all format in K562 leukemia cells. The CDKO screen has a completely different library structure than Big Papi and is from an independent institution. Since comprehensive validation of genetic interactions in CDKO does not exist, we used SynLethDB (Guo et al. Nucleic Acids Research, 2016), an extensive database containing synthetic lethal pairs collected from a variety of data sources. Treating SynLethDB interactions as non-cell line specific, we found 90 synthetic lethal pairs common between SynLethDB and CDKO, which we used as a true positive set and in parallel we constructed a set of 207 true negative pairs using negative control guides. Given these validation sets, we calculated the ROC-AUC and PR-AUC for GEMINI, GImap, and π-score. We could not run dLFC as there is no implementation/code available. GEMINI outperformed GImap and π-score, where our ROC-AUC and PR-AUC were 0.08 and 0.12 higher than GImap and 0.3 and 0.48 higher than π-score. We have added a new subsection to the manuscript (Impact of library design on performance), as well as Figure S10 in Additional file 1 to benchmark our method on data from CDKO . “GEMINI requires each guide to be paired with at least one biological negative control guide to initialize the CAVI approach”.*

2.It appears that the GEMINI approach will have a special requirement for the design of CRISPR screen, and this raises a question whether GEMINI is generally applicable for CRISPR combination screens.

*We acknowledge that this may appear to be a limitation in terms of the scope of our approach. However, in all published combinatorial screens to date and in multiple ongoing screens that we are aware of, each guide is paired with at least one negative control. Nonetheless, in all-by-all format screens, if negative controls are not provided, GEMINI can use the median LFC values for a single gene paired with all other genes to initialize the inference procedure. To specifcally test this, we performed comparisons in both the CDKO and Big Papi datasets to assess the difference in performance when a negative control is or is not specified. In the case of Big Papi data, GEMINI shows a significant increase in performance when a negative control is provided (ROC-AUC improvement of 0.15 and PR-AUC improvement of 0.1, Figure S11a in Additional file 1). In the CDKO data, GEMINI performs robustly whether or not a negative control is specified, but still demonstrates an improvement using negative control (ROC-AUC improvement of 0.05 and PR-AUC improvement of 0.07, Figure S11b in Additional file 1). Thus, GEMINI provides additional flexibility, as all existing approaches are dependent on the inclusion of negative control guides.*

*We have modified the manuscript (Impact of library design on performance) and added Figure S11 in Additional file 1 to address this concern.*

3. Another concern is the comparisons with other methods. AUC curves between different methods are shown only on this “Big Papi” dataset, but not on other public datasets.

*As described in the first comment, we have now addressed this by benchmarking our method on the CDKO dataset as well, and continue to show that GEMINI outperforms existing approaches.*

Minor comments

1. The definition of “sensitive lethality and recovery” is hard to understand (page 9, line 31). What does it mean to rank relationships based on min(yg,l; yh,l) − yg,l − yh,l − sg,h,l?

*We apologize for the confusion in our definition of sensitive and strong interactions. To clarify, the strong score system represents genetic interactions with high synergy, where the combination effect of an interaction (i.e. |sg,h,l|) is much greater than either of the individual effects (i.e. max(|yg,l|, |yh,l|)). This can also be thought of as interactions with a phenotype that is “much more than additive”. On the other hand, the sensitive score best captures interactions where the phenotype of the pair provides increased lethality or recovery compared to either gene individually. Specifically, in the case of “sensitive” lethality, we compare the total effect of gene pairs g and h (i.e. yg,l + yh,l + sg,h,l) to the most lethal individual gene (i.e. min(yg,l, yh,l)). Hence, we compute this as min(yg,l, yh,l) − (yg,l + yh,l + sg,h,l). Note that we also impose a condition such that neither gene g or h should be completely lethal by itself (i.e. yg,l & yh,l > cλ). This scoring system will capture the additional dropout that results from a gene pair, where the killing effect of the pair cannot be attributed to the knockout of either gene individually. Here, the calculated scores are used to rank the relative strengths of interactions in each sample, and the highest scores correspond to the most synergistic lethal interactions.*

*We have expanded on our descriptions of these scoring systems both in the “Results” and “Methods” sections of the manuscript.*

2. It is important to plot the normalized read count changes for the reported gene pairs to make sure the pairs are real in the screen.

*Thank you for this point. We have now added plots showing the normalized read counts of early vs. late time points for gene pairs reported in Fig. 2b in A549 (Figure S3 in Additional file 1), as well as for common interactions found across cell lines in Fig. 3a Figure S5 in Additional file 1). These additions indicate that GEMINI's findings are in agreement with the normalized read counts.*
